# Novel Combinatorial MicroRNA-Binding Sites in AAV Vectors Synergistically Diminish Antigen Presentation and Transgene Immunity for Efficient and Stable Transduction

**DOI:** 10.3389/fimmu.2021.674242

**Published:** 2021-04-28

**Authors:** Manish Muhuri, Wei Zhan, Yukiko Maeda, Jia Li, Anoushka Lotun, Jennifer Chen, Katelyn Sylvia, Ishani Dasgupta, Motahareh Arjomandnejad, Thomas Nixon, Allison M. Keeler, Sangeetha Manokaran, Ran He, Qin Su, Phillip W. L. Tai, Guangping Gao

**Affiliations:** ^1^ Horae Gene Therapy Center, University of Massachusetts Medical School, Worcester, MA, United States; ^2^ Department of Microbiology and Physiological Systems, University of Massachusetts Medical School, Worcester, MA, United States; ^3^ VIDE Program, University of Massachusetts Medical School, Worcester, MA, United States; ^4^ Department of Medicine, University of Massachusetts Medical School, Worcester, MA, United States; ^5^ Department of Pediatrics, University of Massachusetts Medical School, Worcester, MA, United States; ^6^ Li Weibo Institute for Rare Diseases Research, University of Massachusetts Medical School, Worcester, MA, United States

**Keywords:** adeno-associated virus vectors, microRNA, miR-BS, miR-142, miR-652-5p, miR-223-3p, antigen presenting cells, gene therapy

## Abstract

Recombinant adeno-associated virus (rAAV) platforms hold promise for *in vivo* gene therapy but are undermined by the undesirable transduction of antigen presenting cells (APCs), which in turn can trigger host immunity towards rAAV-expressed transgene products. In light of recent adverse events in patients receiving high systemic AAV vector doses that were speculated to be related to host immune responses, development of strategies to mute innate and adaptive immunity is imperative. The use of miRNA binding sites (miR-BSs) to confer endogenous miRNA-mediated regulation to detarget transgene expression from APCs has shown promise for reducing transgene immunity. Studies have shown that designing miR-142BSs into rAAV1 vectors were able to repress costimulatory signals in dendritic cells (DCs), blunt the cytotoxic T cell response, and attenuate clearance of transduced muscle cells in mice to allow sustained transgene expression in myofibers with negligible anti-transgene IgG production. In this study, we screened individual and combinatorial miR-BS designs against 26 miRNAs that are abundantly expressed in APCs, but not in skeletal muscle. The highly immunogenic ovalbumin (OVA) transgene was used as a proxy for foreign antigens. *In vitro* screening in myoblasts, mouse DCs, and macrophages revealed that the combination of miR-142BS and miR-652-5pBS strongly mutes transgene expression in APCs but maintains high myoblast and myocyte expression. Importantly, rAAV1 vectors carrying this novel miR-142/652-5pBS cassette achieve higher transgene levels following intramuscular injections in mice than previous detargeting designs. The cassette strongly inhibits cytotoxic CTL activation and suppresses the Th17 response *in vivo*. Our approach, thus, advances the efficiency of miRNA-mediated detargeting to achieve synergistic reduction of transgene-specific immune responses and the development of safe and efficient delivery vehicles for gene therapy.

## Introduction

Adeno associated virus (AAV) vector-mediated gene therapies have emerged as the platforms of choice for the treatment of monogenic diseases. First isolated from adenovirus preparations in the 1960s (1, 2), AAV is a non-pathogenic dependoparvovirus that is able to transduce a wide range of cell types. Recombinant AAVs (rAAVs) have been proven to confer long-lasting and safe transgene expression in a variety of human tissues (3–6). rAAVs have also achieved sustained therapeutic effect for a variety of inherited diseases, including Leber’s congenital amaurosis type 2 (7, 8), hemophilia B (9), M-type a-1 antitrypsin deficiency (10, 11), and lipoprotein lipase deficiency (12, 13). Two AAV-based drugs (Luxturna and Zolgensma) to date have been approved by the FDA, and >100 clinical trials using AAV-based therapies are in progress (5, 6, 14, 15).

AAV vectors are known to possess a weak immunological footprint, in part, because of their relative inability to transduce antigen presenting cells (APCs). However, there have been multiple reports of vector-related toxicities that have compromised transgene product expression (3, 16, 17). Human clinical trials have also demonstrated how B and T cell immune responses directed against the AAV capsid, likely arising after natural infection with wild-type AAV, might potentially impact gene transfer safety and efficacy in patients (3). Moreover, AAV-delivered transgene products are often presented as foreign antigens that can stimulate host immune responses, and can lead to the generation of transgene-specific antibodies and cytotoxic T lymphocytes (CTL) (18, 19). The quality and intensity of humoral and cellular immune responses can vary depending on the transgene DNA composition, vector tropism to APCs, route of administration, and the tissue target.

Skeletal muscle is considered an important target tissue for AAV-mediated vector gene transfer. Intramuscular administration of rAAV-encoding therapeutic transgenes enables muscle to serve as a bio-factory for the sustained production of secreted proteins (20–25). Notably, AAV serotype 1 (AAV1) is known for its tropism to skeletal muscle cells and also has a limited system-wide biodistribution profile. Intramuscular administration of rAAV1 in humans has been used previously for the exogenous expression of therapeutic proteins (26–28). However, targeting muscle tissues can result in immune reactions, and anti-transgene responses have been mostly documented in gene therapy trials involving intramuscular delivery of rAAV vectors (28, 29). One of the primary reasons attributed to anti-transgene toxicity is the unwanted transduction of APCs. rAAV transduction of professional APCs, like dendritic cells (DCs), macrophages, and B cells leads to the presentation of transgenic peptides on MHC class I molecules, culminating in cytotoxic T cell-mediated clearance of transfected cells. Importantly, rAAV1 has also been shown to transduce APCs to elicit transgene immunity (30). The use of muscle-specific promoters in rAAV expression cassettes have shown limited success in controlling leaky expression in DCs (31, 32). Alternatively, microRNA (miRNA)-mediated detargeting *via* posttranscriptional control has been successfully demonstrated to restrict cell type-specific transgene expression with lentiviral gene transfer to the mouse liver (33, 34).

Detargeting transgenes from specific cell types *via* endogenously expressed miRNAs can also be used to enhance tissue-specificity by excluding spurious transgene expression from non-target cells (35–39). miR-142 is regarded as a hematopoietic-specific miRNA and is expressed at high levels in APCs (33, 40). In the absence of miR-142, DCs show reduced production of proinflammatory cytokines and the ability to activate T cells in mice (41). We have previously shown that miR-142-mediated APC detargeting boosts transgene levels and inhibits antibody formation and blunts the cytotoxic T cell response (42). Incorporation of two or three miR-142 binding sites achieved detargeting from APCs to levels that enable sufficient stable transgene expression (30, 42) following intramuscular injections. However, CD8+ T cell infiltrates were still observed at early treatment timepoints (two weeks post-injection), suggesting that full APC detargeting and maximal transgene expression may not have been achieved with miR-142BS cassettes alone.

In this study, we have identified two miRNAs, miR-223-3p and miR-652-5p, whose expression is enriched in immune cell populations in mice. miR-652 and miR-223 are expressed in cells of the myeloid lineage, including monocytes and granulocytes (43). Incorporation of binding sites for miR-223-3p and miR-652-5p, in combination with miR-142, can effectively detarget expression of the chicken ovalbumin (OVA) transgene from APCs following intramuscular administration. The novel combinatorial microRNA-binding site (miR-BS) designs effectively improve transgene expression, blunt antibody response against the transgene, and reduce the activation of T cells. Furthermore, the miR-142/652-5pBS cassette confers the lowest capacity for triggering OVA-specific cytotoxic CTL activation and inhibits the activation of Th1 and Th17 cells more effectively than miR-142BS on its own. This unique miR-BS design therefore confers a global immunosuppressive milieu that is specific to the transgene. Our findings not only reiterate the therapeutic potential of miRNA-mediated detargeting cassettes, but also demonstrate that a combination of different miR-BSs might have an additive or synergistic effect on inhibition of transgene immunity.

## Materials and Methods

### Vector Plasmid Construction and rAAV Production

rAAV expression cassette was made by inserting full-length OVA cDNA between the chicken β-actin (CB) promoter and rabbit β-globin (RBG) polyA signal to generate the pAAV.*CB.OVA cis* plasmid (42). For pAAV.*CB.OVA*.miR-BS constructs, two copies of the miR-BS sequence, individually or in combination with miR-142BS, were inserted between the *OVA* cDNA and *RBG* polyA signal. The sequences of the miR-BS are listed in **Table 1**. All expression cassettes were verified by Sanger sequencing. rAAV1 vectors were produced by the Viral Vector Core at the University of Massachusetts Medical School as previously described (42, 65, 66).

**Table 1:** List of candidate miRNA binding sites shortlisted for *in vitro* screening.

miRNA	Cell types enriched with miRNAs	Sequences of binding sites	References
miR-106	Monocyte	(CTACCTGCACTGTTAGCACTTTG)_2_	(44, 45)
miR-126a	pDC	(CGCATTATTACTCACGGTACGA)_2_	(46, 47)
miR-142	DC	(TCCATAAAGTAGGAAACACTACA)_2_	(40, 41, 48)
miR-16	B	(CGCCAATATTTACGTGCTGCTA)_2_	(49, 50)
miR-17	B, T, Monocyte	(CTACCTGCACTGTAAGCACTTTG)_2_	(45)
miR-18	B, T, Monocyte	(CTATCTGCACTAGATGCACCTTA)_2_	(44, 51)
miR-19a	B, T, Monocyte	(TCAGTTTTGCATAGATTTGCACA)_2_	(44, 51)
miR-19b	B, T, Monocyte	(TCAGTTTTGCATGGATTTGCACA)_2_	(44, 51)
miR-20	B, T, Monocyte	(CTACCTGCACTATAAGCACTTTA)_2_	(44, 52)
miR-21a	B, Monocyte, MF	(TCAACATCAGTCTGATAAGCTA)_2_	(53)
miR-223	Myeloid	(TGGGGTATTTGACAAACTGACA)_2_	(43, 54)
miR-24-3p	DC	(CTGTTCCTGCTGAACTGAGCCA)_2_	(48)
miR-29a	T	(TAACCGATTTCAGATGGTGCTA)_2_	(55, 56)
miR-29b	T	(AACACTGATTTCAAATGGTGCTA)_2_	(55)
miR-29c	T	(TAACCGATTTCAAATGGTGCTA)_2_	(55)
miR-302a-3p	MF	(TCACCAAAACATGGAAGCACTTA)_2_	(57, 58)
miR-30b	DC	(AGCTGAGTGTAGGATGTTTACA)_2_	(48)
miR-33-5p	MF	(TGCAATGCAACTACAATGCAC)_2_	(59)
miR-34a	B, DC	(ACAACCAGCTAAGACACTGCCA)_2_	(53)
miR-424	Monocyte	(TTCAAAACATGAATTGCTGCTG)_2_	(60, 61)
miR-652-3p	DC	(AATGGCGCCACTAGGGTTGTG)_2_	(43)
miR-652-5p	DC	(GAATGGCACCCCCTCCTAGGGTTG)_2_	(43)
miR-9-3p	MF	(ACTTTCGGTTATCTAGCTTTAT)_2_	(62, 63)
miR-9-5p	MF	(TCATACAGCTAGATAACCAAAGA)_2_	(61, 62)
miR-92a	B, T, Monocyte	(CAGGCCGGGACAAGTGCAATA)_2_	(45)
miR-99b-5p	MF, DC	(CGCAAGGTCGGTTCTACGGGTG)_2_	(52, 64)

### *In Vitro* Screening of OVA Constructs

OVA expression plasmids with or without the miR-142BS elements were transfected into mouse myoblast C2C12 cells (ATCC, CRL-1772) and the macrophage cell line RAW264.7 (ATCC, TIB-71) using jetPRIME transfection reagents (Polyplus-transfection SA) according to the manufacturer’s instructions. C2C12 and RAW264.7 cells were cultured in Dulbecco’s modified Eagle medium (Hyclone, SH30022) with 20% and 10% fetal bovine serum, respectively (FBS, Hyclone, SH30071), and 1% penicillin/streptomycin (Hyclone, SV30010). C2C12 cells were differentiated by culturing the cells in DMEM containing 2% horse serum (HyClone) and 1 μM insulin (Sigma-Aldrich). Mouse dendritic cells (JAWS II; ATCC, CRL-11904) were cultured in α minimum essential medium (MilliporeSigma, M8042) with ribonucleosides, deoxyribonucleosides, 4 mM L-glutamine, 1 mM sodium pyruvate, and 20% FBS with 5 ng/mL murine GM-CSF. JAWS II were transfected by Nucleofection. Briefly, 2.0 × 10^6^ cells were collected and resuspended in 100 μL Nucleofector Solution (Lonza, V4XP-4024) at room temperature. Plasmids were then added, mixed, and transferred into Nucleocuvette Vessels. The P4 HF program for immature mouse DCs was selected and ran. Then, 2 mL of medium was added, and cells were split into a 24-well plate (500 μL/well) (Corning, CLS3527). Three days after transfection, supernatants were collected for OVA ELISA. A Gaussia luciferase expression plasmid was transfected along with OVA expression plasmids to account for transfection variabilities. Transfections were done in triplicate for each round.

### Mice

C57BL/6 mice were purchased from The Jackson Laboratory and maintained at the University of Massachusetts Medical School. Mice were housed under specific pathogen-free conditions. Six- to eight-week-old male mice were injected unilaterally into tibialis anterior (TA) muscles with 1.0 × 10^11^ genome copies (GCs) of rAAV1 diluted in sterile phosphate-buffered saline (PBS). Blood samples were collected *via* facial vein by using an animal lancet (Goldenrod) and BD Microtainer tubes with serum separator additive (Becton Dickinson and Company). All animal procedures were approved by the Institutional Animal Care and Use Committee of the University of Massachusetts Medical School. Experiments were conducted in accordance with relevant guidelines and regulations.

### ELISAs

Serum levels of OVA and anti-OVA IgG were determined by ELISA. Briefly, 96-well Nunc Maxisorp Immunoplates (Thermo Fisher Scientific) were coated with 2 μg/mL of rabbit anti-OVA polyclonal antibodies (AB1225, MilliporeSigma) or OVA protein (MilliporeSigma) in 100 μL coating buffer (KPL) per well. After an overnight incubation at 4°C, plates were washed with 0.05% Tween-20 in PBS, followed by incubation with blocking buffer (KPL) for two hours at room temperature. For OVA detection, the samples were diluted 100-fold with ELISA diluent (KPL), and OVA protein standards (Bioworld) were two-fold serially diluted with 1% normal mouse serum starting from 50 ng/mL. Then, 100 μL of sample or standard was added to plates and incubated for one hour at room temperature. After washing four times, peroxidase-conjugated rabbit anti-OVA polyclonal antibody (200-4333-0100, Rockland Immunochemicals) (1:5,000 diluted) was added and incubated for one hour at room temperature. For anti-OVA IgG1 detection, samples were diluted 1:200, and the mouse anti-OVA IgG1 (sc-80589, Santa Cruz Biotechnology) was used as the standard. After a one-hour incubation in OVA-coated plates, wells were washed, HRP-conjugated goat anti-mouse IgG1 (sc-2060, Santa Cruz Biotechnology) was added, and plates were incubated for another hour at room temperature. Plates were then washed four times and incubated with 100 μL of ABTS HRP Substrate (KPL). Optical density at 410 nm was measured using a Synergy HT microplate reader (BioTek). Standard curves for OVA and IgG1 were generated by using the 4-parameter logistic regression with Gen5 software (BioTek).

ELISA quantification of secreted cytokine levels was performed using customized ProcartaPlex Immunoassays (Thermo Fisher Scientific) following manufacturer’s instructions. Briefly, the samples were incubated in a 96-well plate (Corning) with magnetic beads conjugated to antibodies against desired cytokines for two hours at room temperature with shaking. Wells were then washed thrice with wash buffer, using a magnetic plate washer (Bio-Rad). This step was followed by incubation with detection antibody for one hour at room temperature with shaking. Following three washes, the samples were incubated with Streptavidin-PE for 30 mins at room temperature with shaking. The samples were finally resuspended in 1X reading buffer after three washes. Plates were read in a MAGPIX^®^ System instrument (Luminex Corporation). Standard curves were generated, and the levels of each cytokine were calculated using the 4-parameter logistic regression using GraphPad Prism 8.

### Isolation of Immune Cells From Liver and TA Muscle

Mice were anesthetized and perfused with PBS by transcardial perfusion. Livers and injected TA muscles were harvested from perfused mice and stored temporarily in RPMI media on ice. Tissues were minced with a razor blade followed by enzymatic digestion (0.4% Collagenase type II (Sigma-Aldrich) and 300 μg/mL DNase I (Millipore Sigma) for 30 min at 37°C. Dissociated livers were strained through a 70 μm cell strainer (Falcon) and washed twice with 1X processing buffer (5% FBS in PBS). Cell pellets were resuspended in 40% Percoll (GE Healthcare) and carefully overlaid onto 70% Percoll followed by centrifugation for 25 mins at 400 g, with the brakes off. Leukocytes that band at the 40-70% interphase are removed with a pipette onto a fresh tube and washed thrice with 1X processing buffer to prepare them for staining.

Minced TAs were incubated with 0.5 mg/mL DNase I and 0.25 mg/mL Liberase TL (Roche) in processing buffer for two hours at 37°C. The digested pieces were pooled and strained through a 70 μm cell strainer. Cell suspensions were washed at 1,500 rpm for 7 min in complete RPMI followed by resuspension of the cell pellet in processing buffer for staining.

### Flow Cytometry

Cells were suspended in 100 μL PBS with 5% FBS and washed once in PBS. For live/dead staining, cells were resuspended in PBS containing Fixable viability dye eFluor 506 (Thermo Fisher Scientific; 1:1000 dilution) and incubated for 30 min at 4°C in the dark. Following one wash with FACS buffer (2% FBS in PBS), the cells were blocked with anti-CD16/32 (2.4G2) mAb (BD Biosciences, catalog 553141; 1:100 dilution) for 15 min at 4°C. After blocking, the corresponding antibodies were added at 1:100 dilution for 30 min at 4°C in the dark. Following antibody staining, cells were washed twice in FACS buffer. Flow cytometry analyses were performed on an Attune NxT Flow Cytometer (Thermo Fisher Scientific). Data were analyzed using FlowJo (Tree Star).

For intracellular staining, cells were permeabilized in a 1X solution of fixation/permeabilization solution (BD Biosciences) for 30 min at 4°C after blocking/cell surface staining. Thereafter, the cells were washed thrice in 1X Perm/Wash buffer (BD Biosciences). Antibody dilutions (1:100) are prepared in 1X Perm/Wash buffer and cells were resuspended in the antibody containing solution and incubated for 30 min at 4°C. Following antibody staining, cells were washed once in 1X Perm/Wash buffer and resuspended in FACS buffer for analyses by flow cytometry. The list of antibodies used for staining are provided in **Supplementary Table 3**.

### qPCR and RT-qPCR

Mouse tissue DNA was isolated using the QIAamp genomic DNA kit (QIAGEN) following manufacturer’s instructions. Detection and quantification of vector genomes in extracted DNA were performed by real-time qPCR as described previously (67, 68). Total RNA was isolated from mouse tissues or cells using Trizol (Life Technologies). cDNA preparation for miRNA quantification was done using TaqMan™ MicroRNA Reverse Transcription Kit (Thermo Fisher Scientific) following manufacturer’s instructions. qPCR to quantify expression levels of miR-142-3p, miR-652-3p, miR-652-5p, miR-223-3p, and miR-33-5p were done using TaqMan™ Fast Advanced Master Mix (Thermo Fisher Scientific). Real-time qPCR was performed using the ViiA 7 real-time PCR system (Life Technologies). All other reagents, primers, and probes were purchased from Life Technologies.

### Immunohistochemistry

Mouse tissues were fixed in 10% buffered formalin (Fisher Scientific, catalog SF100-20) overnight and embedded in paraffin. Sections (8 μm thick) were stained with H&E. Images were acquired on a TissueFAXS Whole Slide Scanning System (TissueGnostics) using a 20x objective. Nuclei quantification was performed with Image J.

For immunofluorescence staining, muscle sections were de-paraffinized in xylene and rehydrated using a graded ethanol series culminating with PBS. Following antigen retrieval using a programmable pressure cooker with “target retrieval solution”, pH 6.0 (Dako), tissue sections were blocked with 10% goat serum in PBS. The slides were then stained for CD8 (1:500, D4W2Z, Cell Signaling Technology), granzyme B (1:40, AF1865, R&D Systems), F4/80 (1:100, MCA497R, Bio-Rad), and OVA (1:500, AB1225, Millipore Sigma) for 16 hours at 4°C. Species-specific secondary antibodies conjugated to Cy5 or Cy7 fluorophores were used and incubated for one hour at room temperature in the dark. Sections were washed, counterstained with DAPI (100 ng/ml) and mounted using FluorSave (Calbiochem) mounting medium. Images were acquired on a Leica SP8 laser scanning confocal microscope using a 40x oil-immersion objective. Quantification of the fluorescent signals of the respective markers was performed using QuPath (69).

### Statistics

All data are shown as mean ± SD. Unpaired Student’s t tests (two-tailed), one-way ANOVA and two-way ANOVA, with or without *post hoc* testing, were calculated using GraphPad Prism 8. Differences were considered significant when *p* values were less than 0.05.

## Results

### *In Vitro* Screening of Candidate miR-BSs in DCs, Macrophages, and Muscle Cells

**Figure 1:**
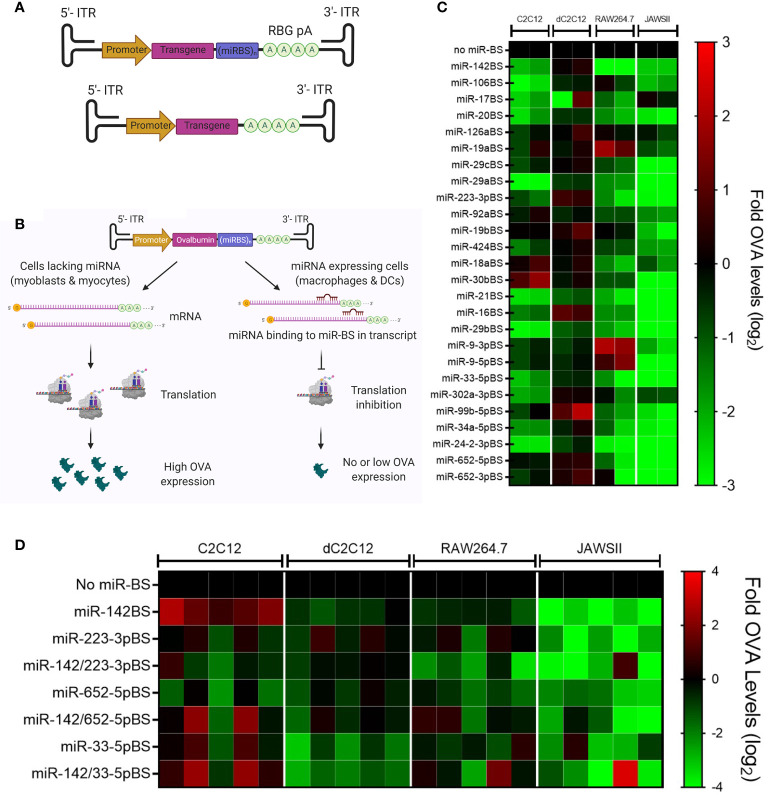
Selection and *in vitro* screening of candidate miRNA binding sites (miR-BS). **(A)** Schematic illustration of rAAV.*OVA* expression vectors. The expression of the OVA transgene is driven by the CB6 promoter. Two copies of miR-BSs are cloned between the transgene and the rabbit β-globin poly A (RBG pA). The OVA expression cassette is flanked by inverted terminal repeats (ITRs) at both ends. **(B)** An ideal miR-BS candidate, upon delivery to myocytes and myoblasts, which do not express the corresponding miRNA, is expected to undergo transcription and translation to produce high levels of transgene. In contrast, when this vector is delivered to miRNA-expressing immune cells, like macrophages and DCs, miRNA binding to the transgene mRNA leads to translational inhibition and transcript degradation, resulting in minimal transgene protein production. **(C)** Summary of the *in vitro* screening of individual miR-BS candidates represented as a heat map with OVA expression denoted as fold change (log_2_) with respect to expression vectors lacking any miR-BS. **(D)** The *in vitro* screening of miR-BS combinations summarized as a heat map with relative OVA levels represented as fold change (log_2_) (*n* = 5). C2C12, mouse myoblasts; dC2C12, mouse myocytes; RAW264.7, mouse macrophages; JAWSII, mouse DCs.

To perform functional validation of miR-BS-mediated detargeting, two copies of miR-BSs were engineered into the 3′-UTR of the highly immunogenic chicken OVA cDNA. OVA is used as a model immunogen for studying antigen-specific immune responses in mice. The occurrence of immune responses against the OVA transgene, and the resulting loss of OVA protein expression, following intramuscular delivery by rAAVs have been described previously by us and others (30, 42). Transgene expression is driven by the strong and ubiquitous CMV enhancer/chicken β-actin promoter to achieve ubiquitous transcription, irrespective of cell type (70). These expression cassettes were then subcloned into rAAV vectors (**Figure 1A**). In the absence of publicly available databases that display cell type-specific miRNA expression, a list of candidate miRNAs whose expression levels were reported in the literature to be enriched in hematopoietic lineage cells (DCs, monocytes, B and T cells) was generated. Two copies of binding sites for these miRNAs were individually cloned into the rAAV expression cassettes to generate a library of 26 vectors (**Table 1**).

The inhibitory effects of individual miR-BSs on OVA expression was first evaluated in mouse immature DCs (JAWS II), mouse macrophage cells (RAW264.7), and the mouse C2C12 skeletal myoblast cell line. Expression plasmids were transfected into the aforementioned cell types along with a *Gaussia* luciferase (GLuc) expressing plasmid to account for transfection variability. The conditioned media from the transfected cells were harvested 72 hours post-transfection for measuring secreted OVA levels by ELISA. The levels of OVA were then normalized to GLuc levels for each transfection. Transfected C2C12s were also differentiated under serum starvation conditions to examine the effects of the miR-BS in myocytes (dC2C12). An ideal miR-BS candidate is expected to retain high OVA expression in myoblasts and myocytes indicating specific transgene expression in muscle cell types, but exhibit reduced expression in DCs and macrophages, reflecting translational inhibition in APCs (**Figure 1B**). The results of the *in vitro* screen revealed that the miR-142BS element in dC2C12s conferred levels of OVA expression that were equal to those conferred by the construct that lacks miR-BSs, and significantly reduced OVA levels in JAWSII and RAW264.7 cells (**Figure 1C**). This is consistent with previously reported studies where miR-142BSs were shown to successfully detarget rAAV-delivered transgenes from APCs and to suppress anti-transgene immunity in mice (30, 42). We also note that despite the fact that APCs are enriched with these miRNAs as described in the literature, the design of some cognate miR-BS, namely miR-126aBS and miR-19aBS, failed to reduce transcript expression in JAWSII and RAW264.7 cells. These results demonstrate that not all 3’-UTR modifications were capable of reducing transcript stability.

### Combinatorial miR-BS Designs in rAAV-OVA Vectors Increase Transgene Expression With Negligible Anti-OVA Antibody Responses

Several miR-BS candidates maintained high muscle expression of OVA while conferring detargeting from immune cells, albeit to slightly lesser degrees than what was achieved by miR-142BS. Three of these: miR-223-3pBS, miR-652-5pBS, and miR-33-5pBS were further selected for combinatorial designs with miR-142BS. Two copies of each miR-BS were cloned along with two copies of miR-142BS in the 3’-UTR of the OVA transgene. The resulting miR-BS expression vectors were then screened for OVA expression in JAWSII, RAW264.7, and C2C12 cells. The most promising miR-BS combinations to emerge from this round of screening were miR-142/223-3pBS and miR-142/652-5pBS (**Figure 1D**).

**Figure 2:**
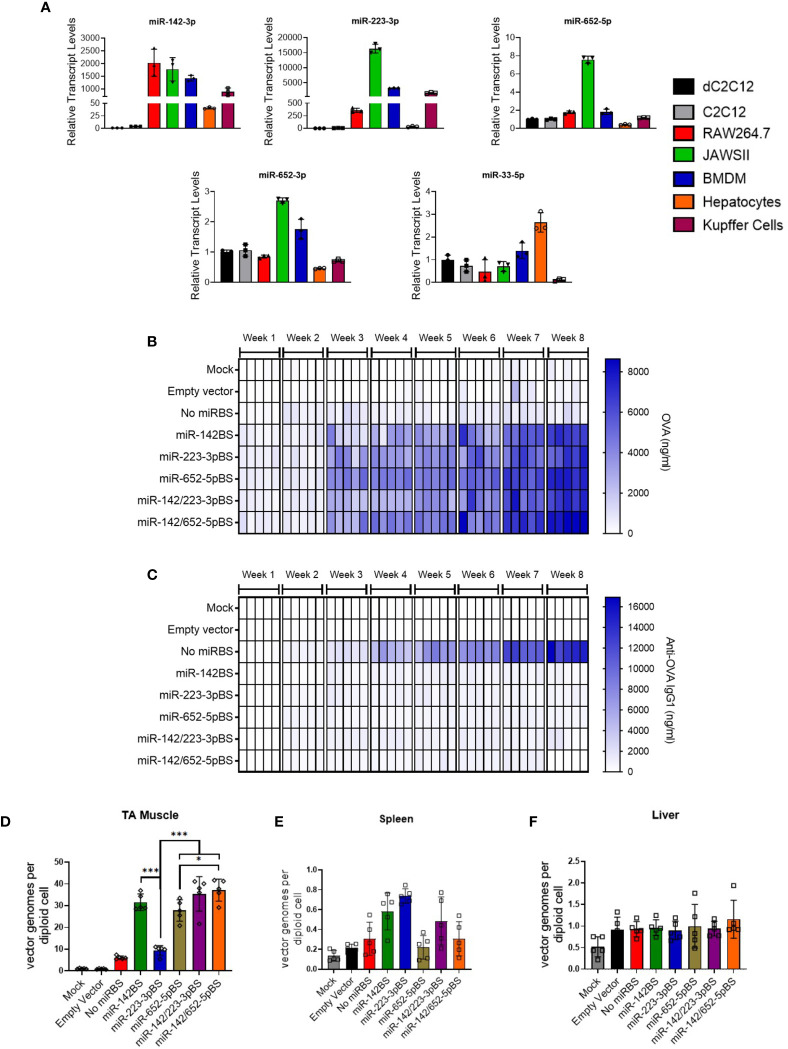
Incorporation of miR-223BSs and miR-652BSs boosts *in vivo* OVA production and suppresses antibody development. **(A)** Endogenous miRNA expression levels in cultured mouse myoblasts (C2C12), myocytes (dC2C12), macrophages (RAW264.7), DCs (JAWSII), bone marrow derived macrophages (BMDM), primary mouse hepatocytes, and Kupffer cells as quantified by reverse transcription quantitative PCR (RT-qPCR) (n = 3). rAAV1 expression vectors were injected by i.m. on day 0 followed by serum collection every week for an eight-week period. **(B**, **C)** ELISA quantification of circulating OVA expression **(B)** and anti-OVA IgG1 **(C)** (1 × 10^11^ GCs/mouse, *n* = 10). Single gradient heat map representing respective analyte levels (*n* = 5). **(D–F)** ddPCR detection of rAAV vector genome copies in injected skeletal muscle **(D)**, spleen **(E)**, and liver **(F)** at eight weeks post-injection (*n* = 5). Values represent mean ±SD. *p < 0.05, ***p < 0.001, one-way ANOVA with Tukey’s *post hoc* test.

To provide support for the notion that the miR-BS cassettes are operating through endogenously expressed cognate miRNAs, we quantified the levels of these miRNAs in cells from immunological and non-immunological lineages to demonstrate their levels of enrichment in APCs. Interestingly, miR-223-3p was found to be approximately 500- to 15,000-fold higher in cell types of the immunological lineage (RAW264.7, JAWSII, bone marrow derived macrophages (BMDM), and Kupffer cells) than in C2C12 and differentiated C2C12 cells (**Figure 2A**). Expression of miR-652-5p in RAW264.7 and JAWSII cells was about 2- and 8-fold higher than levels observed in C2C12 cells, respectively. On the other hand, no significant enrichment of miR-652-3p and miR-33-5p was seen in immune cell types (**Figure 2A**).

We next aimed to assess the function of miR-223-3p and miR-652-5p binding sites *in vivo*. We therefore packaged the rAAV-OVA expression cassettes into AAV1 capsids with or without the individual miR-223-3p or miR-652-5p binding sites, or in combination with miR-142BS elements. Produced vectors were then injected into TA muscles of adult mice. Mice administered with rAAV1 empty capsids or PBS (mock) were used as controls. Animals injected with rAAV1.*OVA*.miR-BS vectors generated increasingly high and sustained levels of OVA expression in circulation, with a negligible anti-OVA antibody response (IgG1). In contrast, animals treated with rAAV1.*OVA* without miR-BSs showed baseline levels of OVA after eight weeks (**Figures 2B, C**). These animals also generated the highest levels of anti-OVA antibodies, which were substantially greater than the anti-OVA IgG levels produced in mice injected with rAAV1.OVA.miR-BS vectors (**Figures 2B, C** and **Supplementary Tables 1, 2**). Interestingly, the combination of *miR-142* and *652-5p* binding sites (miR-142/652-5pBS), or miR-652-5pBS alone, conferred the highest serum OVA levels. The differences in OVA expression levels between this combination and other miR-BS designs were most pronounced at eight weeks post-injection (*p* <0.001) (**Supplementary Figure 1A** and **Supplementary Table 1**). However, anti-OVA antibody production in mice injected with any of the miR-BS expressing vectors was not significantly different from each other, indicating that incorporation of any individual or combination of the tested miR-BSs led to similar levels of anti-transgene antibody suppression (**Supplementary Figure 1B** and **Supplementary Table 2**). With the exception of miR-223-3pBS, the levels of vector genomes detected in TAs eight weeks after injection were high (**Figure 2D**). Interestingly, there was a greater than five-fold increase in the abundance of vector genomes in muscles treated with miR-BS-containing vectors, than in muscles treated with the rAAV-*OVA* construct that lacks miR-BSs (**Figure 2D**). Consistent with immune clearance of transduced muscle fibers and loss of vector genomes, rAAV1.*OVA*.miR-142/652-5pBS-transduced muscle tissues showed at least a six-fold increase in vector genomes at eight weeks as compared to rAAV-*OVA* with no miR-BSs (**Figure 2D**). As expected, vector genome counts in the spleen and liver were at near background levels of detection (**Figures 2E, F**).

### miR-142BS and Other Novel miR-BS Designs Downregulate Macrophage Activation and Costimulatory Signals in DCs

Although there were no clear differences in the anti-OVA antibody levels conferred between the miR-BSs cassette designs, we wondered whether any underlying immune responses against the vector and/or the transgene product might still preclude efficient OVA transduction, which can be overcome by optimizing APC detargeting. To address this notion, we analyzed immune effector cell activation following vector treatment. Mice injected intramuscularly with rAAV1 vectors with or without miR-BSs were sacrificed at four weeks post-injection and cells were isolated from injected TAs.

**Figure 3:**
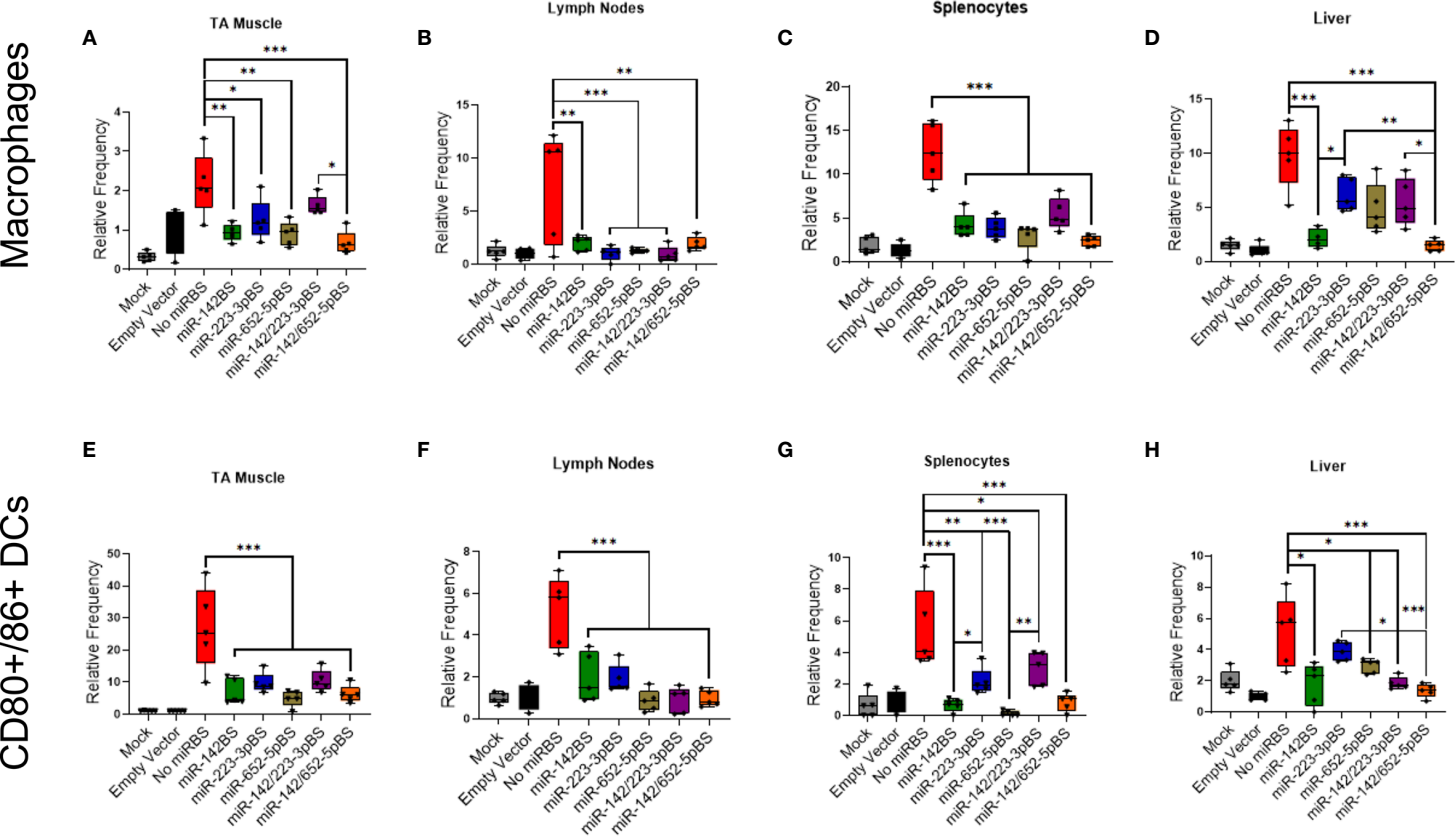
Incorporation of miR-142BS, miR-223BS, and miR-652BS into rAAV1.*OVA* transgene vectors reduces macrophage and DC activation. rAAV1.*OVA* expressing vectors with or without miR-BSs (1 × 10^11^ GCs/mouse) were delivered by i.m. injections into C57BL/6 mice. Four weeks after injection, cells were isolated from TAs, lymph nodes, spleens, and livers and stained for macrophage markers (CD11b+, F4/80+) and activated DCs (CD11c+, CD80+, CD86+) followed by flow cytometry analysis. Relative frequencies of macrophage populations **(A–D)** and activated DCs **(E–H)** are represented as box plots with means, first and third quartile boundaries, and whiskers indicating max and min values. (*n* = 5). Mock = AAV1.empty capsid. *p* values were determined by one-way ANOVA with Tukey’s *post hoc* test. *p < 0.05, **p < 0.01, ***p < 0.001.

The antigen-specific T cell receptor (TCR) binds foreign peptide antigen-MHC complexes, and the CD28 receptor binds to B7 (CD80/CD86) costimulatory molecules expressed on the surface of APCs, a process that is vital to initiating and maintaining the proliferation of T cells (71). Immunophenotyping of isolated cells by flow cytometry revealed an overall depletion of macrophages and CD80/CD86-positive DCs (CD11c+ cells) in mice injected with vectors carrying miR-BS at the four-week time point (**Figures 3A, E**). The greatest repression was achieved with vectors harboring the miR-652-5pBS or miR-142/652-5pBS cassettes. Notably, there was also a remarkable decrease in overall activated DCs in TA muscles across different vectors (**Supplementary Figure 2A**).

Lymph nodes are secondary lymphoid organs where different immune cell populations coordinate both the innate and adaptive arms of the immune response. Therefore, we also examined the number of CD80/CD86-positive DCs in draining lymph nodes of the injected limb at two and four weeks post-injection. We observed a significant reduction in the population of activated macrophages, CD80/CD86-positive DCs in the animals treated with vectors carrying miR-BSs (**Figures 3B, F** and **Supplementary Figure 2B**, **Supplementary Figures 3A–C**). However, the suppression in macrophage and DC activation did not significantly vary among the different miR-BS designs.

In our previous report, we demonstrated that miR-142BS-mediated APC detargeting leads to a reduction of co-stimulatory molecule expression in isolated splenocytes (42). To further confirm this effect, we isolated splenocytes from injected mice and stained them for macrophage, DC, and DC co-stimulatory markers. All miR-BS-containing vectors significantly suppressed DC activation, macrophage activation, and CD80/86-positive DCs in splenocytes (**Figures 3C, G** and **Supplementary Figure 2C**). While most miR-BS designs mediated weak suppression at two weeks post-injection, indicating no change in the activation state of macrophages and DCs, miR-142BS-, miR-652-5pBS-, and miR-142/652-5pBS-containing vectors inhibited CD80/86 expression as early as two weeks following administration (**Supplementary Figures 4A–C**).

We also isolated immune cells from the livers of injected mice and found that miR-142/652-5pBS-containing vectors mediated the strongest reduction of activated macrophages, DCs, and CD80/86-positive DCs (**Figures 3D, H** and **Supplementary Figure 2D**).

Finally, to determine the activation status of circulating immune cells, we immunophenotyped peripheral blood lymphocytes (PBLs) isolated from the blood of treated mice four weeks post-injection. We did not observe any differences in the levels of activated macrophages, DCs, and CD80/86-positive DCs in the presence of miR-BSs (**Supplementary Figures 5A–C**); indicating that immune cell activation occurred within different tissue compartments, not systemically.

### miR-BS-Mediated APC Detargeting Downregulates OVA-Specific T Cell Activation

**Figure 4:**
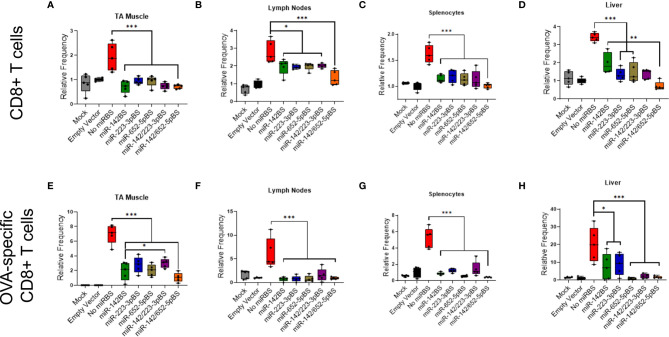
miR-142BS-, miR-223BS-, and miR-652-mediated detargeting suppresses OVA specific CD8 T cell response. C57BL/6 male mice, six weeks old, were i.m. injected with mock, empty capsid, or rAAV1.*OVA* vectors with or without miR-BSs (1 × 10^11^ GCs/mouse). Mice were sacrificed four weeks after treatment and cells were isolated from TAs, lymph nodes, spleens and livers. Cells were then stained for CD8 T cell markers or with anti-CD8a/H-2Kb SIINFEKL tetramer and quantified by flow cytometry (*n* = 5). Relative frequencies of CD8+ T cells **(A–D)** and OVA-specific CD8+ T cells **(E–H)** are depicted as box plots with means, first and third quartile boundaries, and whiskers indicating max and min values (*n* = 5). *p* values were estimated by one-way ANOVA with Tukey’s *post hoc* test. *p < 0.05, **p < 0.01, ***p < 0.001.

We previously established that miR-142BS-mediated APC detargeting achieves circumvention of adaptive immunity by blunting OVA-specific CD8+ T cell response, resulting in sustained transgene expression (42). To assay the ability of rAAV1.*OVA*-miR-BS vectors to engage the adaptive immune response, recruitment of CD4+ and CD8+ T cells was measured four weeks following vector administration in the injected TA muscles, lymph nodes proximal to the injection site, the spleen, and the liver. Analyses of the overall CD8+ T cell populations showed that all of the tested miR-BS designs significantly repressed CD8+ T cell response to the vector in all tissues (**Figures 4A–D**).

Although CD8+ T cell responses in tissues were significantly repressed with groups treated with vectors harboring miR-BS cassettes, they were not indicative of the transgene-specific CD8+ T cell activation status. We therefore assessed whether there was any reduction in CD8+ T cell response specific to OVA protein. The ovalbumin SIINFEKL peptide fragment is recognized by the MHC class I molecule (H-2Kb) of T cells in mice. Therefore, OVA-specific CD8+ T cells can be identified by staining cells with H-2Kb/SIINFEKL MHC Tetramers and quantified by flow cytometry. At four weeks post-injection, we observed that the levels of activated OVA-specific CD8+ T cells were reduced by all miR-BS designs. Notably, vectors carrying miR-652-5pBS and miR-142/652-5pBS appeared to confer the strongest reduction (**Figures 4E–H**). The extent of reduction in lymph nodes and splenocytes were nearly equal to those conferred by PBS and empty capsid treatments. Interestingly, the miR-142BS and miR-223-3pBS cassettes were not as sufficient as other miR-BS designs in reducing OVA-specific CD8+ T cell responses in the liver (**Figure 4H**). Furthermore, reduced activation of CD8+ T cells and OVA-specific CD8+ T cells was observed in lymph nodes and spleens as early as two weeks post-injection (**Supplementary Figures 3D, E** and **Supplementary Figures 4D, E**), but not in TA muscle and liver (data not shown). Significant differences in OVA-specific CD8+ T cells were not observed between the different miR-BS combinations, except in lymph nodes at two weeks post-injection (**Supplementary Figure 3E**). Immunophenotyping of PBLs at four weeks post-injection revealed a significant reduction in circulating OVA-specific CD8+ T cells, but not overall CD8+ T cells for vectors containing miR-652-5pBS, miR-142/223-3pBS, and miR-142/652-5pBS combinations as compared to the vector without miR-BSs (**Supplementary Figures 5A, B**).

We next tested the effect of vector injection on the CD4+ T cell population. A significant reduction in CD4+ T cell counts was seen in the injected TAs across all vectors containing miR-BSs. Consistent with other cell types, miR-652-5pBS and miR-142/652-5pBS cassettes were the most efficient in suppressing CD4+ T cell activation (**Supplementary Figure 6A**). A similar decrease in CD4+ T cell numbers was also observed in lymph nodes, the spleen, and the liver (**Supplementary Figures 6B–D**). An overall reduction in the CD4+ T cell population in PBLs was also seen in treatment groups receiving vectors containing any combination of miR-BSs with no notable differences when compared to each other (**Supplementary Figure 5C**). Taken together, we were able to ascertain that incorporation of miR-652-5pBS in the rAAV expression cassette mediated efficient suppression of macrophage, DC, CD4+, and CD8+ T cell activation and a decrease in the expression of co-stimulatory markers. In certain cases, this effect was enhanced when miR-652-5pBS was combined with miR-142BS.

### miRBS-Mediated APC Detargeting Downregulates OVA-Specific Th1 Response, Inflammatory Cytokine Production, and Memory T Cells

**Figure 5:**
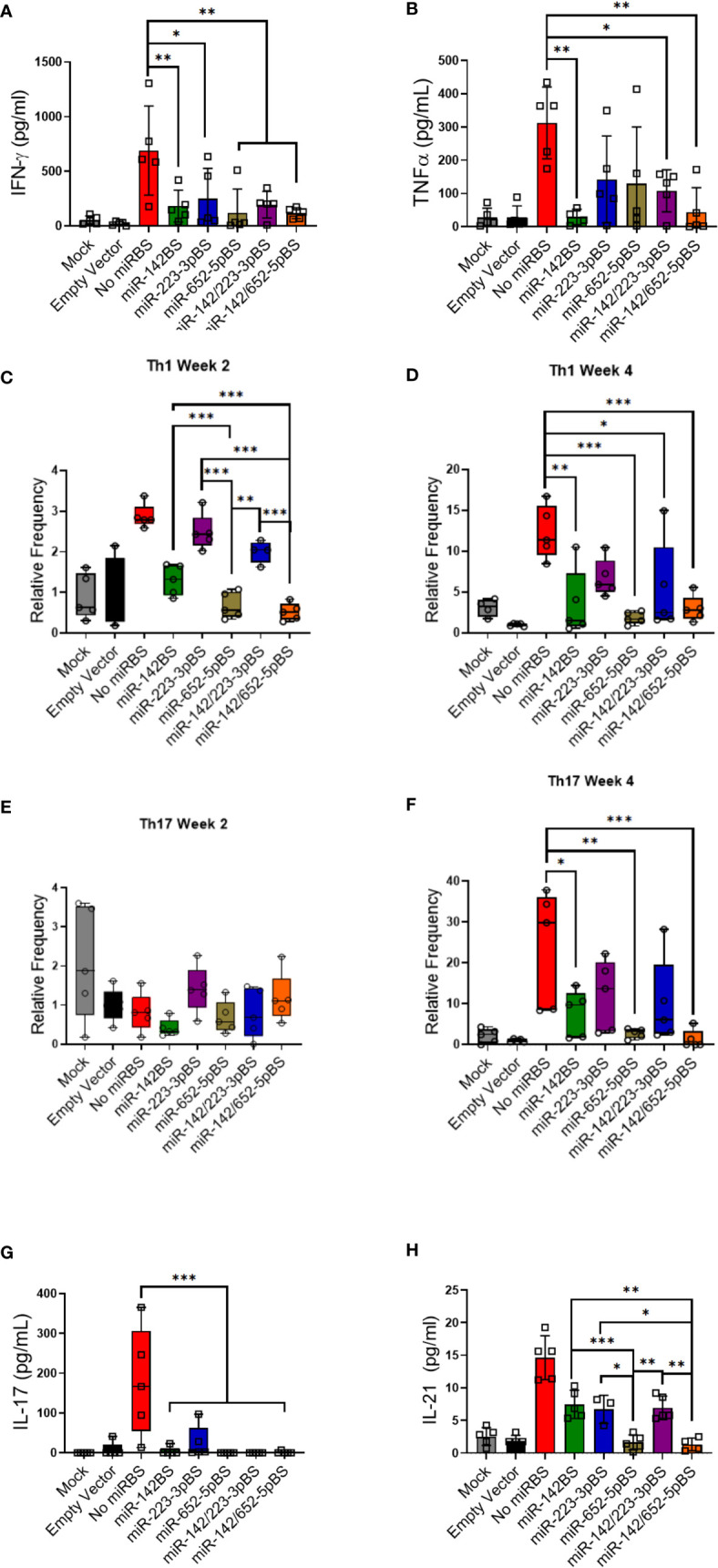
miR-BS incorporation diminishes transgene-specific Th1 and Th17 inflammatory responses. **(A, B)** Estimation of IFN-γ and TNF-α response to OVA stimulation (5 μg/mL) by splenocytes isolated from mice four weeks after vector injection. Three days after treatment, supernatants were collected and quantitated by ELISA (mean ± SD, *n* = 5). *p* values were determined by ANOVA with Tukey’s *post hoc* test. Splenocytes isolated two and four weeks post-AAV1 injection were stimulated for 24 hours with OVA (5 μg/mL) and stained for detecting either Th1 population (CD4+) **(C, D)** or Th17 cell population (CD4+IL-17A+) **(E, F)** and analyzed by flow cytometry (Box plots with means, first and third quartile boundaries, and whiskers indicating max and min values; *n* = 5). **(E, F)** Levels of secreted Th17-specific cytokines, IL-17 and IL-21- were assessed after stimulation of splenocytes (harvested four weeks post-injection) for 72 hours with OVA (5 μg/mL) by ELISA (mean ± SD, *n* = 5). *p < 0.05, **p < 0.01, ***p < 0.001. *p* values were estimated by one-way ANOVA with Tukey’s *post hoc* test.

Previous studies have shown that TNF-α and IFN-γ are two principal pro-inflammatory cytokines produced in response to rAAV transduction (72). TNF-α is produced by DCs and other immune cells and is involved in both innate and adaptive immune responses (71, 73). IFN-γ is the classic cytokine secreted by Th1 cells and promotes phagocytosis and upregulates microbial killing. We therefore sought to determine TNF-α and IFN-γ response to APC-detargeted vectors. We isolated and cultured splenocytes from mice that were injected with rAAV1.*OVA* vectors with or without miR-BSs at both two- and four-week timepoints. Upon OVA stimulation, splenocytes from mice treated with rAAV1.*OVA* with no binding sites secreted high levels of TNF-α and IFN-γ. In contrast, vectors carrying miR-BSs attenuated cytokine responses to levels that were on average greater than two-fold reduced, which is comparable to cytokines secreted by the splenocytes from mice that received PBS and empty capsids (**Figures 5A, B**). To further validate the suppression of Th1 response, the OVA stimulated splenocytes were stained for IFN-γ producing CD4 T cells and analyzed by flow cytometry. OVA-specific Th1 cell counts were high in rAAV1.*OVA* splenocytes as early as two weeks post-injection (**Figures 5C, D**). However, the reductions in the Th1 response became significant at two and four weeks post-injection only when miR-142BS, miR-652-5pBS, or both elements were incorporated into vectors. Interestingly, splenocytes from rAAV1.*OVA*.miR-223-3pBS-treated mice showed no change at week 2 in IFN-γ-producing Th1 cells as compared to splenocytes from rAAV1.*OVA-*treated animals. Addition of miR-142BS to these vectors reduced responses to an extent at week 2 (**Figure 5C**). Interestingly, miR-223-3pBS-containing vectors (in combination with or without miR-142BS) seems to completely suppress Th1 activation by week 4 (**Figure 5D**). This may suggest a possibility that the suppression of immune cell activation mediated by miR-223-3pBS incorporation follows kinetics that are slower than those conferred by miR-652-5pBS-mediated suppression.

The effects of miR-BS APC detargeting was also gauged by the activation of CD4+ and CD8+ memory T cells. In mice, CD4 and CD8 T cells can be further categorized into memory and naïve phenotypes, based on CD62L (L-selectin) and CD44 expression. CD44^low^CD62L+ populations are considered naïve (T_N_) cells, CD44^high^CD62L+ populations are considered central memory (T_CM_) cells, and the CD44^high^CD62L^neg^ populations are considered effector and/or effector memory (T_E/EM_) cells. It is known that CD4 and CD8 T cells differ in their distribution of these subsets in lymphoid and peripheral organs (74, 75). We therefore evaluated OVA-stimulated splenocytes for CD4 and CD8 memory T cell activation (both T_CM_ and T_EM_) at two weeks post-rAAV injection. While there was a substantial reduction in T_CM_ and T_EM_ populations among CD4+ and CD8+ T cells, with incorporation of miR-652-5pBS and miR-142/652-5pBS elements, no differences were noticed in the naïve T cell population. This may suggest that the phenotypic changes observed in T cell populations can be attributed to the different extents of OVA-specific activation by different vector designs, and not due to the naïve T cell populations that were not stimulated by OVA (**Supplementary Figure 7**). However, the reduction in T_CM_ and T_EM_ populations was not maintained at four weeks post-injection (data not shown).

### The miR-142/652-5pBS Combination Effectively Suppresses Th17 Response

One pathway that lacks study with respect to rAAV transgene immunogenicity is the involvement of Th17 cells. Th17 cells are a recently discovered cell type that secrete IL-17 as their primary effector cytokine and belong to the CD4+ T cell family (76, 77). We hypothesized that these pro-inflammatory cells might be involved in mounting an anti-transgene immune response (78, 79). To investigate their contribution towards anti-transgene immunity, OVA-stimulated splenocytes at two and four weeks post-injection were quantified for IL-17A expressing CD4+ T cells by flow cytometry. While no OVA-specific Th17 response was observed at two weeks, rAAV1.*OVA* splenocytes showed elevation in the number of Th17 cells at four weeks (**Figures 5E, F**). With the exception of miR-223-3pBS, inclusion of the candidate miR-BSs in vectors significantly downregulated Th17 activation, with maximal repression imparted by miR-652-5pBS and miR-142/652-5pBS. This outcome was similar in fashion to Th1 responses under these treatments. Both IL-17 and IL-21 are Th17 cell-secreted cytokines that accentuate the protective effects of Th17-mediated immune response. Therefore, Th17 activation was further confirmed by measuring IL-17 and IL-21 production from OVA-stimulated splenocytes. Consistent with the flow cytometry data, splenocytes from mice treated with rAAV1.*OVA* lacking miR-BSs produced high levels of IL-17. In contrast, vectors carrying miR-BSs conferred a significant reduction in Th17 activation, and hence a concomitant decrease in IL-17 production (**Figure 5G**). Incorporation of miR-652-5pBS and miR-142/652-5pBS seemed to significantly suppress IL-21 secretion in stimulated splenocytes as well (**Figure 5H**). Our data thus suggests that transgene-specific Th17 response might play a critical role in the suppression of transgene expression over time. Incorporation of miR-BSs in expression cassettes blunts this response, and in turn, boosts the levels of transgene expression.

### miRNA-Mediated Detargeting Does Not Activate Regulatory T Cells to Enable Immunosuppression

**Figure 6:**
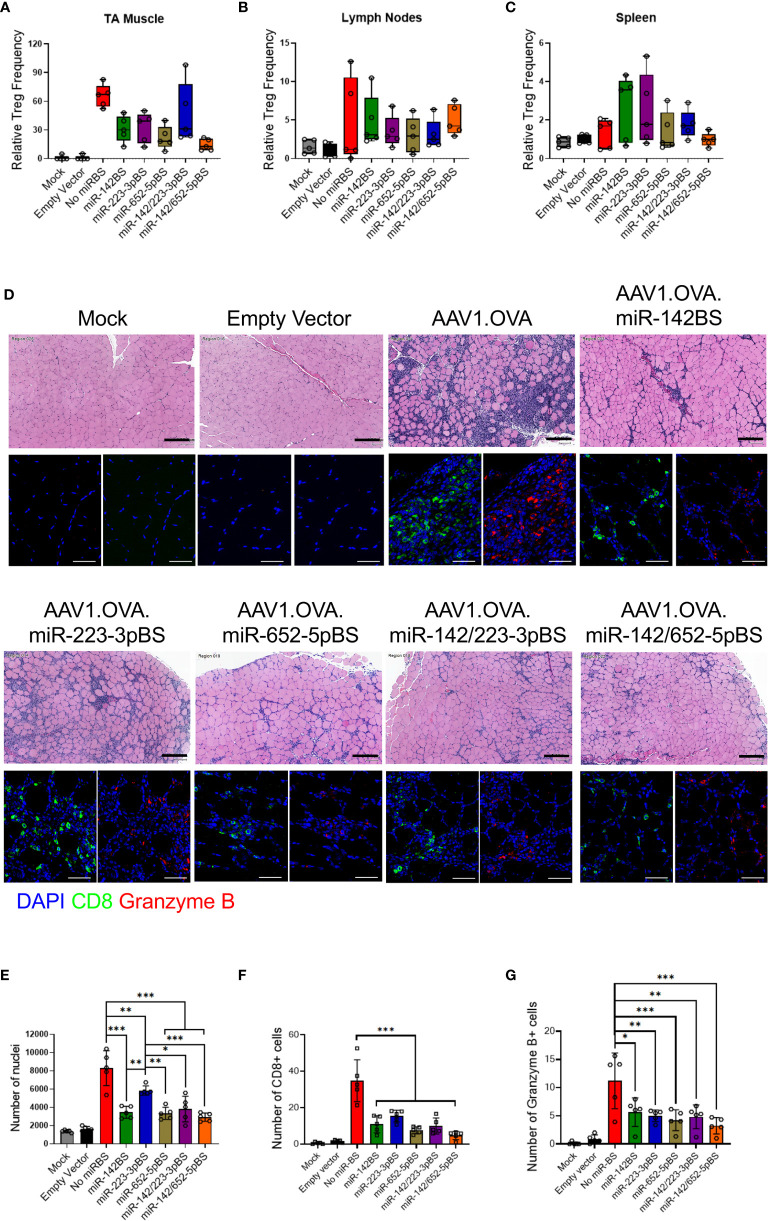
miRNA-mediated detargeting acts independent of Treg immunosuppression and reduces tissue clearance by downregulation of OVA-specific CTL response. **(A–C)** Cells from TA muscle, lymph nodes and spleen were harvested from rAAV1 injected C57BL/6 mice four weeks after treatment and stained for Treg markers (CD4+, CD25+, FOXP3+). The frequencies of Tregs were quantified by flow cytometry and displayed as box and whisker plots (*n* = 5). **(D)** C57BL/6 male mice, six weeks old, were i.m. injected with rAAV1.OVA vectors with or without miRBS (1 × 10^11^ GCs/mouse, *n* = 5) and sacrificed two weeks after injection to harvest muscles. Tissue sections were stained for H&E (upper panels, original magnification: 20x), CD8 and granzyme B (lower panels: DAPI, blue; red, granzyme B, red; and CD8, green; original magnification: 40x). Scale bars = 200 μm (H&E images), 50 μm (fluorescence images). **(E)** Quantification of H&E images for nuclear infiltrates in whole tissue section at original magnification of ×20. **(F, G)** Quantification of CD8 **(F)** and granzyme B **(G)** images in four fields at original magnification of 40x. *p* values were estimated by one-way ANOVA with Tukey’s *post hoc* test. *p < 0.05, **p < 0.01, ***p < 0.001.

The use of miR-142BS elements in lentiviral vectors induces immunologic tolerance and activates regulatory T cells (Tregs) (80). To investigate if this effect is reproduced in rAAV-delivered transgenes containing miR-BSs, we isolated immune cells from TAs, lymph nodes, and spleens at two and four weeks post-injection and stained them for Treg-specific markers. The Treg population can be identified as CD4+ T cells that are also double-positive for CD25 and FOXP3. None of the miR-BS containing vectors lead to an increase in the Treg cell numbers in any of the analyzed tissues (**Figures 6A–C** and **Supplementary Figures 8A, B**). Additionally, stimulated splenocytes from treated animals did not reveal any elevation of the anti-inflammatory cytokines IL-10 and TGF-β (**Supplementary Figures 8C, D**). Therefore, the incorporation of miR-BSs into AAV vectors does not induce Treg activation nor induce tolerance by the suppression of other immune cell types as observed with lentiviral vectors.

### miRBS-Mediated Suppression of CD8+ T Cell Response Reduces Clearance of Transduced Cells and Boosts Transgene Expression

Our previous study demonstrated that inclusion of miR-142BS elements in the rAAV transgene cassette reduced infiltration of CD8+ T cells and subsequent clearance of transduced muscle fibers (42). We aimed to determine whether our novel miR-BS cassettes have the same capacity to repress cytotoxic T cell recruitment and tissue clearance. Histopathology and immunohistopathology analyses of the injected TA muscle tissue was performed two weeks after rAAV treatment. H & E imaging showed that rAAV1.*OVA*-injected TAs had a high degree of cellular infiltration. Tissues injected with AAV vectors containing miR-BSs showed reductions in infiltrates (**Figure 6D**; top panels). Quantification of the number of nuclei in TA cross-sections showed significant decreases in cellular infiltration in mice treated with vectors carrying miR-BSs. Notably, the miR-142/652-5pBS cassette conferred the lowest abundance of immune cell infiltrates (**Figure 6E**). Moreover, H&E-stained cross-sections of TA muscles from animals treated with rAAV1.*OVA* and rAAV1.*OVA*.miR-223-3pBS vectors revealed a high degree of centrally located myonuclei. In healthy muscle fibers, myonuclei are located at the periphery of the muscle fiber. Centrally located myonuclei are indicators of myofiber regeneration following damage (81). These results are thus indicative of active clearance of transduced myofibers and muscle turnover. Notably, centrally located nuclei are absent in the TAs of mice treated with vectors carrying the miR-652-5pBS element (**Figure 6E**).

We also performed immunohistochemical staining of the treated muscle sections for CD8+ T cells, granzyme B, F4/80, and OVA. Granzyme B is a marker for activated cytotoxic T cells and F4/80 is a cell surface marker for macrophages. We observed robust CD8+ T cell (CTL) infiltration in muscle samples from rAAV1.*OVA*-injected animals, and about one-third of infiltrates were positive for granzyme B (**Figure 6D**; bottom panels and **Figures 6F, G**). A significantly lower degree of CTL infiltration was observed among muscles injected with vectors bearing miR-142BS, miR-652-5pBS, miR-142/223-3pBS, and miR-142/652-5pBS elements, of which a very small portion expressed granzyme B. While the number of CD8+ T cells was reduced at least three-fold, there was a two-fold reduction observed in granzyme B expression with the incorporation of these miR-BSs (**Figures 6F, G**). Although miR-223-3pBS-containing vectors showed relatively higher numbers of CD8+ T cells, granzyme B expression was considerably lower, indicating reduced CTL activity. CTL infiltration was also accompanied by macrophage infiltration in the injected muscle tissues of rAAV1.*OVA*-treated mice (**Supplementary Figures 9A, B**). Tissues with a high abundance of CTLs and macrophages also have relatively low OVA expression (**Supplementary Figures 9A, B**). Tissues of mice treated with OVA vectors that harbor miR-BS elements express high levels of OVA and confer reduced levels of macrophage activation (~two-fold reduction). Consistent with CTL infiltration, miR-652-5pBS-bearing vectors showed the lowest degree of infiltration into injected TAs (more than four-fold reduction) by macrophages. These findings indicate that inclusion of miR-BS elements in the OVA transgene led to a reduction in cytotoxic CD8+ T cell and macrophage infiltration, resulting in reduced clearance of transduced muscle fibers, and ultimately efficient and stable transgene expression in the tissue.

## Discussion

Owing to their safety and efficacy in preclinical and clinical studies, rAAVs are promising gene therapy vectors for a variety of genetic diseases (5, 6, 82–84). Skeletal muscle is an attractive target tissue for rAAV-mediated gene therapy for neuromuscular diseases, metabolic disorders, and hemophilia (8, 82, 85–87). The easy accessibility of skeletal muscles makes them ideal for vector administration and the extensive vascular blood supply provides an efficient transport system for secreted therapeutic proteins. However, host immune responses against rAAV1-encoded transgene products after intramuscular delivery have been reported to cause the clearance of transduced fibers and loss of transgene expression (88–91). The induction of these immune responses can be attributed to the undesirable transduction of APCs, such as DCs, which lead to transgene expression and antigen presentation on these cell types. These events, in turn, activate T and B cells (31, 92–94). Therefore, the prevention of transgene-specific immune responses is particularly crucial to the success of rAAV gene therapy in muscles.

miRNA-mediated regulation of transgene expression by engineering miR-BSs in rAAV expression cassettes has proven to reduce transgene-specific immune responses. Binding sites against *miR-122*, *miR-199*, *miR-1*, *miR-183*, and *miR-206* can successfully detarget transgene expression from tissues like liver, heart, dorsal root ganglia and skeletal muscles (35, 36, 95–98). We previously demonstrated that miR-142BS elements can blunt CTL activation and can confer sustained transgene expression in transduced mouse TA muscle (42). *miR-142* is a hematopoietic-specific miRNA whose expression levels are high in APCs, which is central to the effectiveness of miR-142BS-mediated detargeting. Little attempt has been made to identify additional miRNAs that can be utilized to repress transgene expression in APCs and restrain immune response activation. In this study, we identified two miRNAs, miR-223-3p and miR-652-5p that are enriched in macrophages and DCs and have the potential for enhancing APC detargeting. Through *in vitro* and *in vivo* screening of individual miR-BSs and in combination with miR-142BS across several cell types, miR-142/223-3pBS and miR-142/652-5pBS were determined to be potent elements in transgene detargeting from APCs.

DCs are the most potent professional APCs that form an essential link between the innate and adaptive immune responses. Due to their unique aptitude for stimulating T cells, activated DCs play a major role in determining immunological outcome (99, 100). Activation of DCs leads to a significant upregulation of MHC and expression of costimulatory molecules, which play crucial roles in initiating or promoting T cell priming and proliferation. These responses are needed for effective immunity (99). Following AAV infection, both conventional dendritic cells (cDCs) and plasmacytoid DCs (pDCs) are required for the cross-priming of CD8+ T cells, while the other APCs seem to be dispensable for this process (101). miR-BS-mediated detargeting hinders MHC-dependent presentation of the transgenic peptides on the surface of DCs by degradation of the mRNA transcript. In the absence of antigen presentation, the ensuing T and B cell activation and the inflammatory responses are suppressed. Our findings with respect to the efficacy of these novel miR-BSs in promoting transgene detargeting would provide flexibility and alternatives in vector design strategies and increase the safety profile of rAAV-based therapeutics.

Durability and memory are important hallmarks of the adaptive immune system that arise from clonal expansion and differentiation of antigen-specific lymphocytes. Memory CD4+ and CD8+ T cells have low activation thresholds and confer protection in peripheral tissues by responding to antigens upon re-encountering them in secondary lymphoid organs (102, 103). Effector memory T cells (T_EM_) migrate to inflamed peripheral tissues and display immediate effector functions. T_EM_ can be stimulated by antigen presented by nonprofessional APCs in a milieu that does not favor stable cell-cell interactions (104, 105). On the other hand, central memory T cells (T_CM_) have no effector function but readily migrate, proliferate, and differentiate into T_EM_ upon antigenic challenge (106). T_CM_ are more sensitive to antigenic stimulation and less dependent on co-stimulation when compared to naïve T cells and thus provide more effective feedback to stimulation from DCs and B cells (75). Here, we have demonstrated for the first time that rAAV-delivered transgenes can increase both CD4+ and CD8+ memory T cell populations (T_CM_ and T_EM_) in spleen. Importantly, we show that the addition of miR-BSs, specifically miR-652-5pBS and miR-142/652-5pBS significantly suppress memory T cell activation. We previously reported that miR-142BS-mediated detargeting can suppress anti-transgene responses following a second dose of vector to enable successful vector re-administration (42). Further research to determine if redosing of rAAV-delivered transgenes can be enhanced by the inclusion of miR-223-3pBS and miR-652-5pBS elements is warranted.

Another frequently overlooked and under-explored cell type of the CD4+ family are Th17 cells. Th17 cells have been implicated in anti-transgene immune responses in certain preclinical and clinical trials (107, 108). An increase in pro-inflammatory Th17 cells was also observed in non-human primates treated with rAAV-delivered human factor IX (*hF.IX*) (109). It has been hypothesized that a skewed Th17/Treg balance towards increased Th17 values results in higher intensities of anti-transgene immune responses (109). Consistent with this idea, an increase in OVA-specific Th17 cell population was seen in rAAV1.*OVA* vectors, but not in vectors containing miR-652-5pBS or miR-142/652-5pBS cassettes in our present study. To investigate the hypothesis that sufficient Treg activity might be induced to offset the pro-inflammatory Th17 activity by miR-BS inclusion, we quantified the Treg population in TA muscle, lymph nodes, and liver. However, we did not find any difference in the activation status of Tregs, nor an increase in anti-inflammatory cytokine secretion. This finding indicated that tolerance to the transgene protein is likely not mediated by Treg activation, but rather by blunting OVA-specific Th1 and Th17 responses, and inhibition of CTL response.

Taken together, we have provided additional lines of evidence and novel mechanistic insights into the immune cell populations involved in rAAV transgene-specific immune responses. The effect of rAAV administration on Th17 population and memory T cells had yet to be documented. Inclusion of miR-BSs seems to inhibit the activation of these immune cell types. miRNA-mediated detargeting is an approach that has been successfully employed to achieve tissue-and cell type-specific expression by restricting spurious transcription conferred by the wide-tropism profiles inherent to contemporary rAAV platforms. *miR-223-3p* and *miR-652-5p* are enriched in cells of the myeloid lineage and hence serve as ideal candidates for miRNA-mediated regulation. Synergistic action of two miR-BSs has been shown for hepatocyte detargeting (110). Our present study is the first attempt at identifying, screening, and investigating the combinatorial effects of miRNAs for transgene detargeting from APCs following intramuscular injections. Although *miR-652-5p*-mediated transgene regulation seems to be more effective from our findings, miR-223-3pBS incorporation is also effective at transgene detargeting. Interestingly, our results indicate that the kinetics of immune cell suppression by miR-223-3pBS elements is slower and starts becoming evident usually four weeks after vector injection. Additional investigation into whether miR-223-3pBS elements can contribute to immunological suppression in later stages of the therapeutic window may further improve APC-detargeting cassette designs. Finally, in the course of this study, we revealed that some miR-BS cassette designs conferred increases in transgene expression in a cell type-specific manner. Unfortunately, the mechanisms by which these elements increased transgene expression *in vitro* was not investigated. Whether some of these 3’-UTR modifications can act to stabilize transcripts is indeed intriguing and begs further investigation.

The safety of these design elements also need to be closely investigated. miR‐652‐5p has been identified as a disease‐associated miRNA that is dysregulated in various pathological processes like esophageal cancer, bladder cancer, osteosarcoma, gastric cancer, and breast cancer (111–116). miR-223-3p expression has also been shown to be aberrant in gastric cancers, osteosarcoma, glioblastoma, squamous cell carcinoma, breast cancer, neuroblastoma, and myocardial infarction (117–123). Their function as cancer-related miRNAs warrants caution as these elements might trigger unintended consequences in related cell types. Nevertheless, it is worth noting that in an earlier report, miR-BS-mediated post-transcriptional detargeting by AAV vectors did not disturb endogenous miRNA profiles (35).

Importantly, combinatorial miR-BS designs is not just used to alleviate the immunogenic effects, they may also be used for multi-tissue detargeting. The expression profiles of multiple microRNAs can be exploited concomitantly to reshape rAAV tropism to achieve tissue specific expression. Given the small size of these miR-BS elements, they can be combined with other targeting strategies to overcome other roadblocks in rAAV transduction. However, transgene immunogenicity represents a major hurdle in the efficacy of rAAV gene therapy. Continued vector engineering efforts are key to expanding rAAV gene therapy to a wider set of human conditions. In conclusion, we report a post-transcriptionally regulated transgene delivery system where the transgene expression is selectively eliminated from APCs by three miRNAs whose expression is enriched in these cell types. We demonstrate that these miRNAs individually or synergistically are capable of blunting transgene-specific immune response and enable sustained transgene expression over time.

## Data Availability Statement

The original contributions presented in the study are included in the article/**Supplementary Material**. Further inquiries can be directed to the corresponding author.

## Ethics Statement

The animal study was reviewed and approved by Institutional Animal Care and Use Committee of the University of Massachusetts Medical School.

## Author Contributions

MM, and WZ executed the flow cytometry workflows and analyses, with initial guidance from KS and AMK, YM, JL, AL, JC, ID, MA, TN, and PT helped with tissue harvests and sample preparation described in the study. SM, RH and QS produced the vectors used in the study; MM and WZ wrote the initial draft of the manuscript. WZ, MM, PT, and GG revised and finalized the manuscript. MM, PT, and GG developed the study design and interpreted the data. PT and GG supervised the study. All authors contributed to the article and approved the submitted version.

## Funding

This work was partially supported by a Sponsored Research Grant from Voyager Therapeutics and supported by grants from the University of Massachusetts Medical School (an internal grant) and by the NIH (R01NS076991-01, 4P01HL131471-02, UG3 HL147367-01, R01HL097088, U19 AI149646-01).

## Conflict of Interest

GG is a scientific co-founder of Voyager Therapeutics, Adrenas Therapeutics, and Aspa Therapeutics, and holds equity in these companies. GG is an inventor on patents with potential royalties licensed to Voyager Therapeutics, Aspa Therapeutics, and other biopharmaceutical companies.

The remaining authors declare that the research was conducted in the absence of any commercial or financial relationships that could be construed as a potential conflict of interest.
