# Supplementary material for: Novel Combinatorial MicroRNA-Binding Sites in AAV Vectors Synergistically Diminish Antigen Presentation and Transgene Immunity for Efficient and Stable Transduction
Source: Front Immunol. 2021 Apr 28;12:674242. doi: 10.3389/fimmu.2021.674242 (PMC8113644; doi:10.3389/fimmu.2021.674242)
Supplement: Supplementary file 1 [file DataSheet_1.pdf]

Novel combinatorial microRNA-binding sites in AAV vectors synergistically diminish antigen presentation and transgene immunity for efficient and stable transduction

Manish Muhuri[1,2,3], Wei Zhan[1,2,3], Yukiko Maeda[1,3,4], Jia Li[1,2], Anoushka Lotun[1], Jennifer Chen[1], Katelyn Sylvia[1,5], Ishani Dasgupta[1,5], Motahareh Arjomandnejad[1,5], Thomas Nixon[1,5], , Allison M. Keeler[1,5], Sangeetha Manokaran[1], Ran He[1], Qin Su[1], Phillip W.L. Tai[1,2,3†] and Guangping Gao[1,2,6†*]

[1]Horae Gene Therapy Center, University of Massachusetts Medical School, Worcester, MA, USA;
[2]Department of Microbiology and Physiological Systems, University of Massachusetts Medical School, Worcester, MA, USA;
[3]VIDE Program, University of Massachusetts Medical School, Worcester, MA, USA;
[4]Department of Medicine, University of Massachusetts Medical School, Worcester, MA, USA;
[5]Department of Pediatrics, University of Massachusetts Medical School, Worcester, Massachusetts, USA;
[6]Li Weibo Institute for Rare Diseases Research, University of Massachusetts Medical School, Worcester, Massachusetts, USA

[†]These authors share senior authorship
*Correspondence: Guangping Gao Guangping.Gao@umassmed.edu

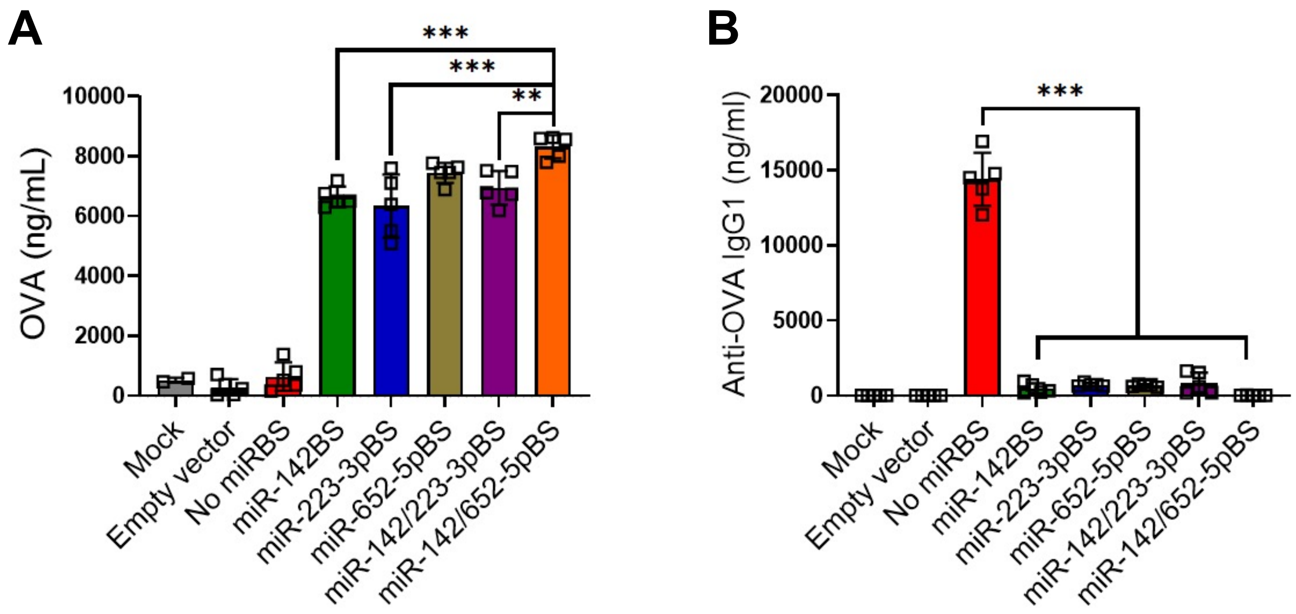

Supplementary Figure 1. The miR-142/652-5pBS cassette is the most efficient at boosting OVA expression and achieves maximal suppression of anti-OVA antibody response. OVA (**A**) and anti-OVA IgG1 (**B**) levels in sera of mice eight weeks after rAAV1 injection estimated by ELISA (mean ± SD, $n = 5$). p values were estimated by one-way ANOVA with Tukey's post hoc test. $^{**}p < 0.01$, $^{***}p < 0.001$.

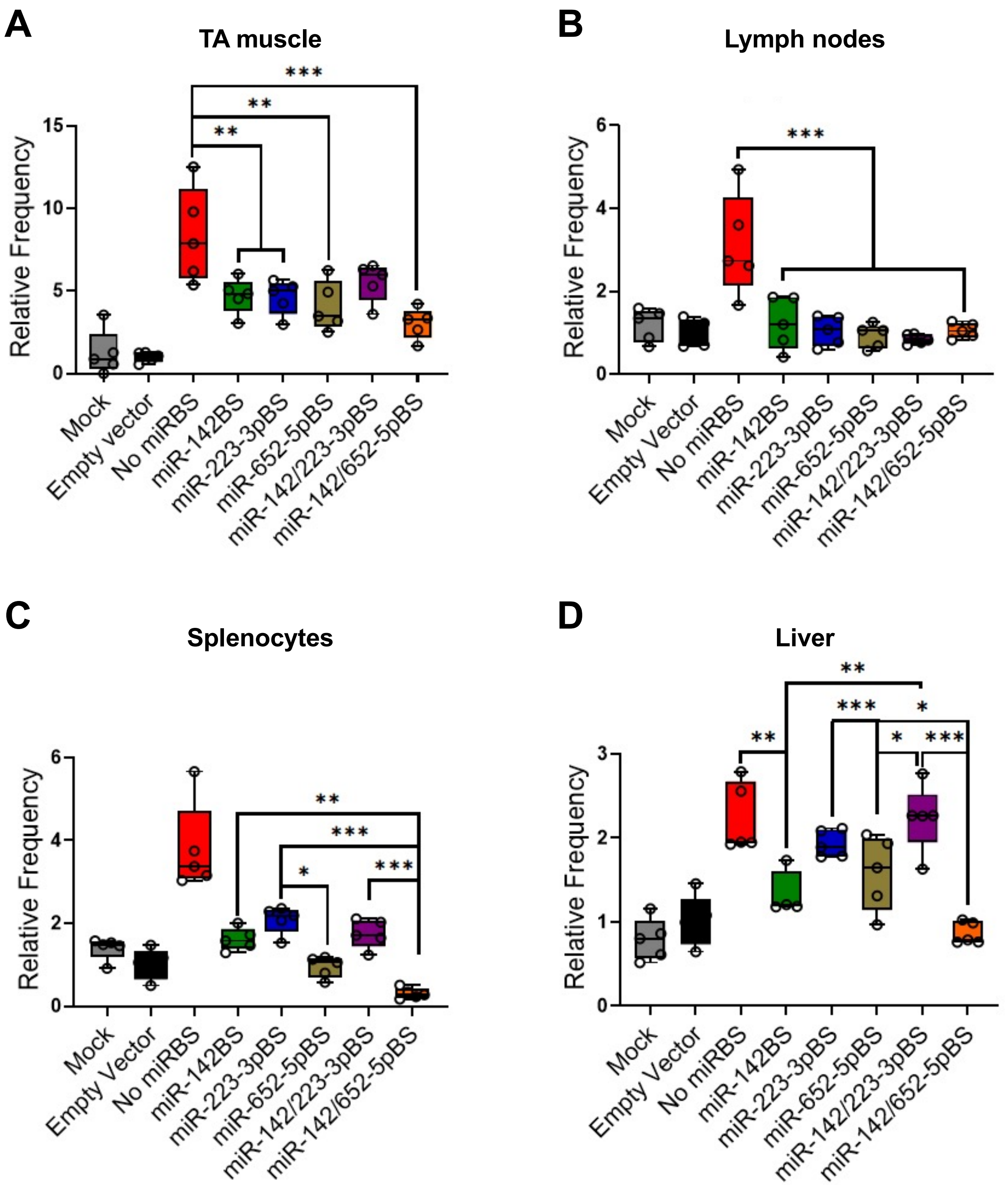

Supplementary Figure 2. miR-BS incorporation mediates robust reduction of DC activation. Six-week-old C57BL/6 male mice were injected by i.m with PBS (mock), empty caspid, rAAV1.OVA, or rAAV1.*OVA*.miR-BS vectors (1×10^{11} GC/mouse, $n = 5$). Quantification of DC populations in TAs (**A**), lymph nodes (**B**), splenocytes (**C**), and livers (**D**) harvested four weeks after vector administration by flow cytometry and represented as box plots with means, first and third quartile boundaries, and whiskers indicating max and min values (n = 5). *p* values were estimated by one-way ANOVA with Tukey's post hoc test. *$p < 0.05$, **$p < 0.01$, ***$p < 0.001$.

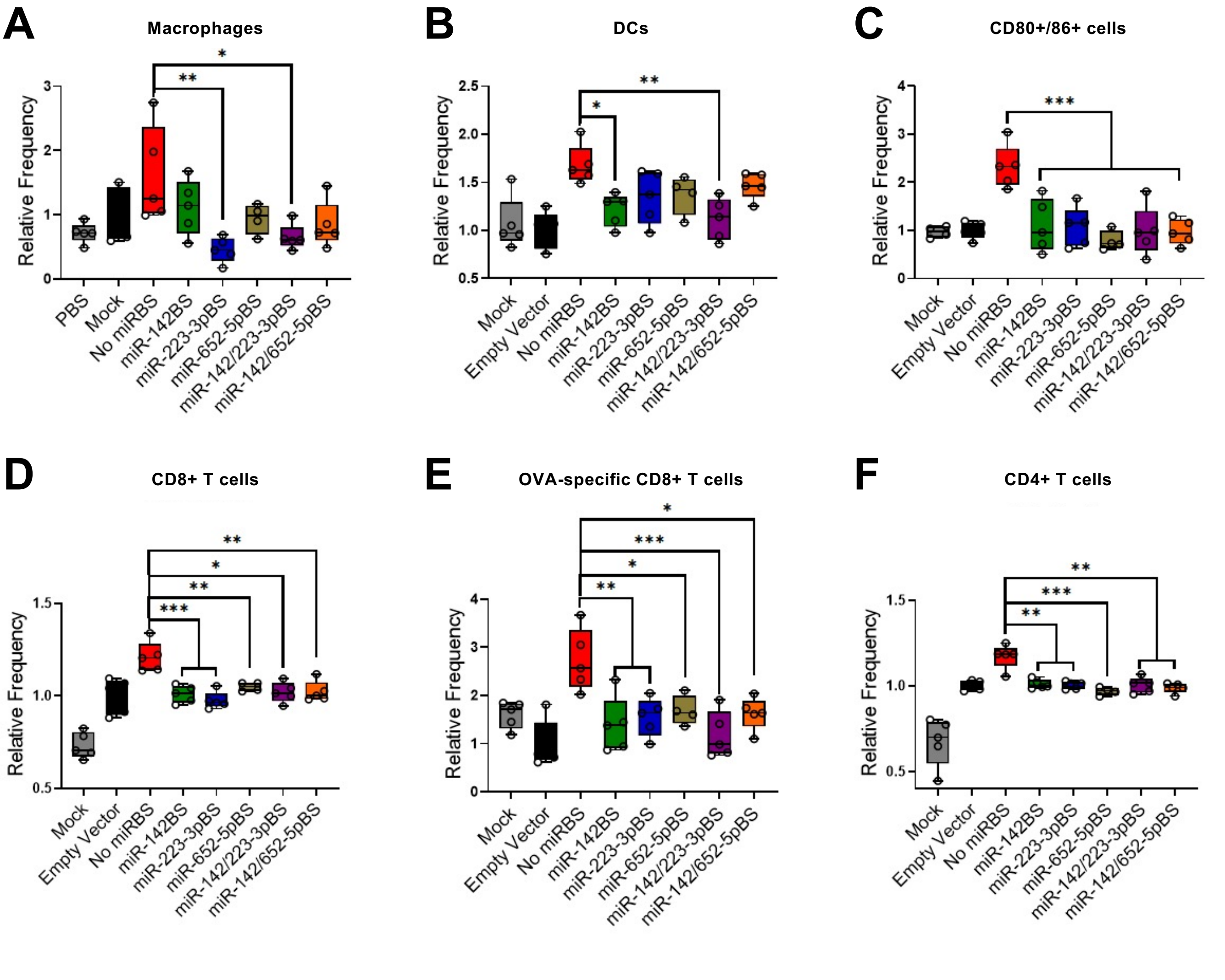

Supplementary Figure 3. miR-BS inclusion in rAAV vectors inhibits activation of pro-inflammatory cells in lymph nodes. Lymph nodes were harvested from C57BL/6 male mice two weeks post-injection with rAAV1 expression vectors. The cells isolated from lymph nodes were stained with antibodies for markers specific to macrophages (**A**), DCs (**B**), activated DCs (**C**), CD8 T cells (**D**), OVA-specific CD8 T cells (**E**), and CD4 T cells (**F**). Relative frequencies of respective immune cell populations were determined by flow cytometry and represented as box plots with means, first and third quartile boundaries, and whiskers indicating max and min values ($n = 5$). p values were estimated by one-way ANOVA with Tukey's post hoc test. *$p < 0.05$, **$p < 0.01$, ***$p < 0.001$.

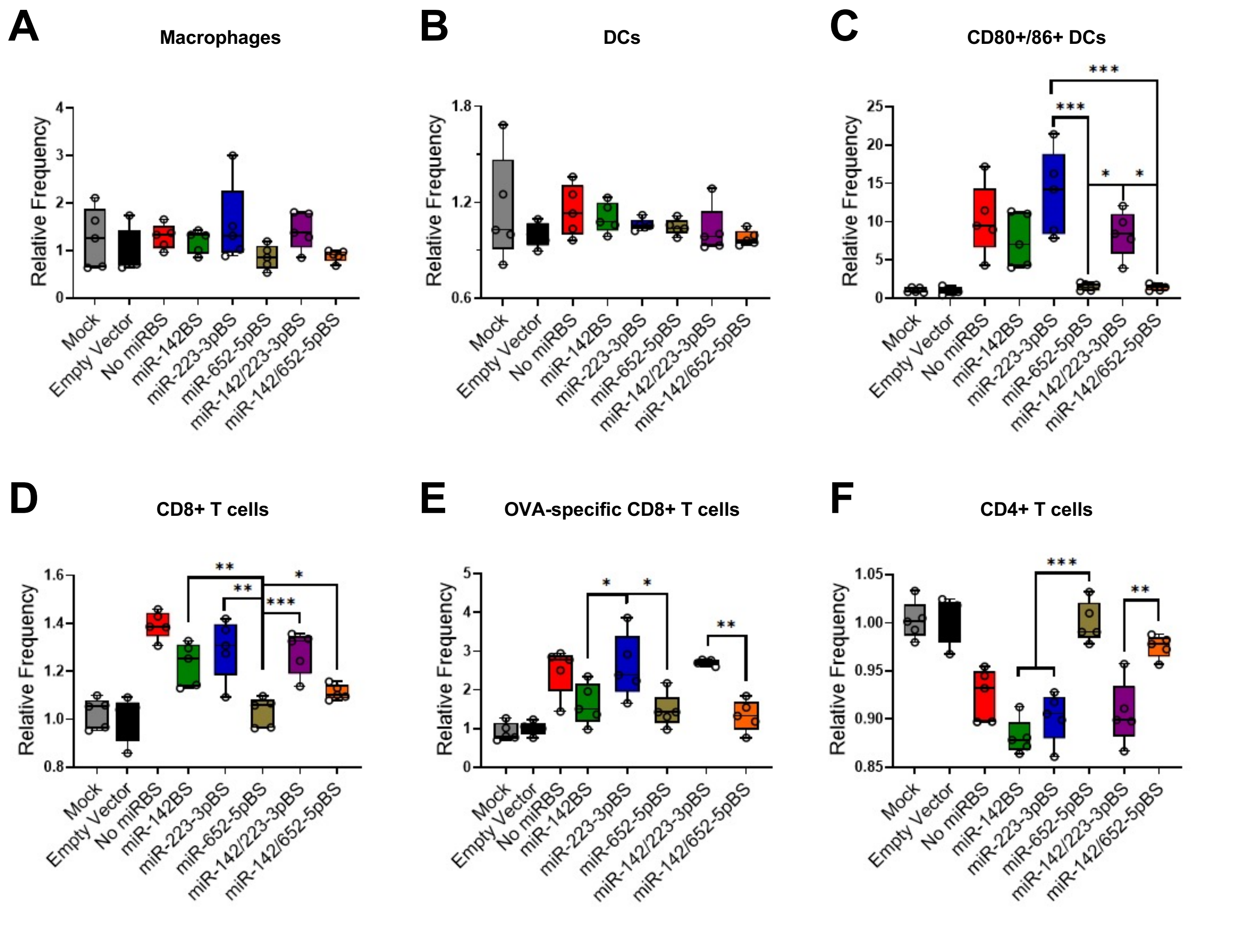

Supplementary Figure 4. Incorporation of miR-142BS and miR-652-5pBS mediates reduction of immune cell activation markers in the spleen. Splenocytes isolated from rAAV-injected mice two weeks post-injection were immunophenotyped to determine activation levels of immune cell populations. Stained cells were analyzed on a flow cytometer and the frequencies of macrophages (**A**), DCs (**B**), activated DCs (**C**), CD8 T cells (**D**), OVA-specific CD8 T cells (**E**) and CD4 T cells (**F**) are displayed as box plots with means, first and third quartile boundaries, and whiskers indicating max and min values (n = 5). p values were estimated by one-way ANOVA with Tukey's post hoc test. *$p < 0.05$, **$p < 0.01$, ***$p < 0.001$.

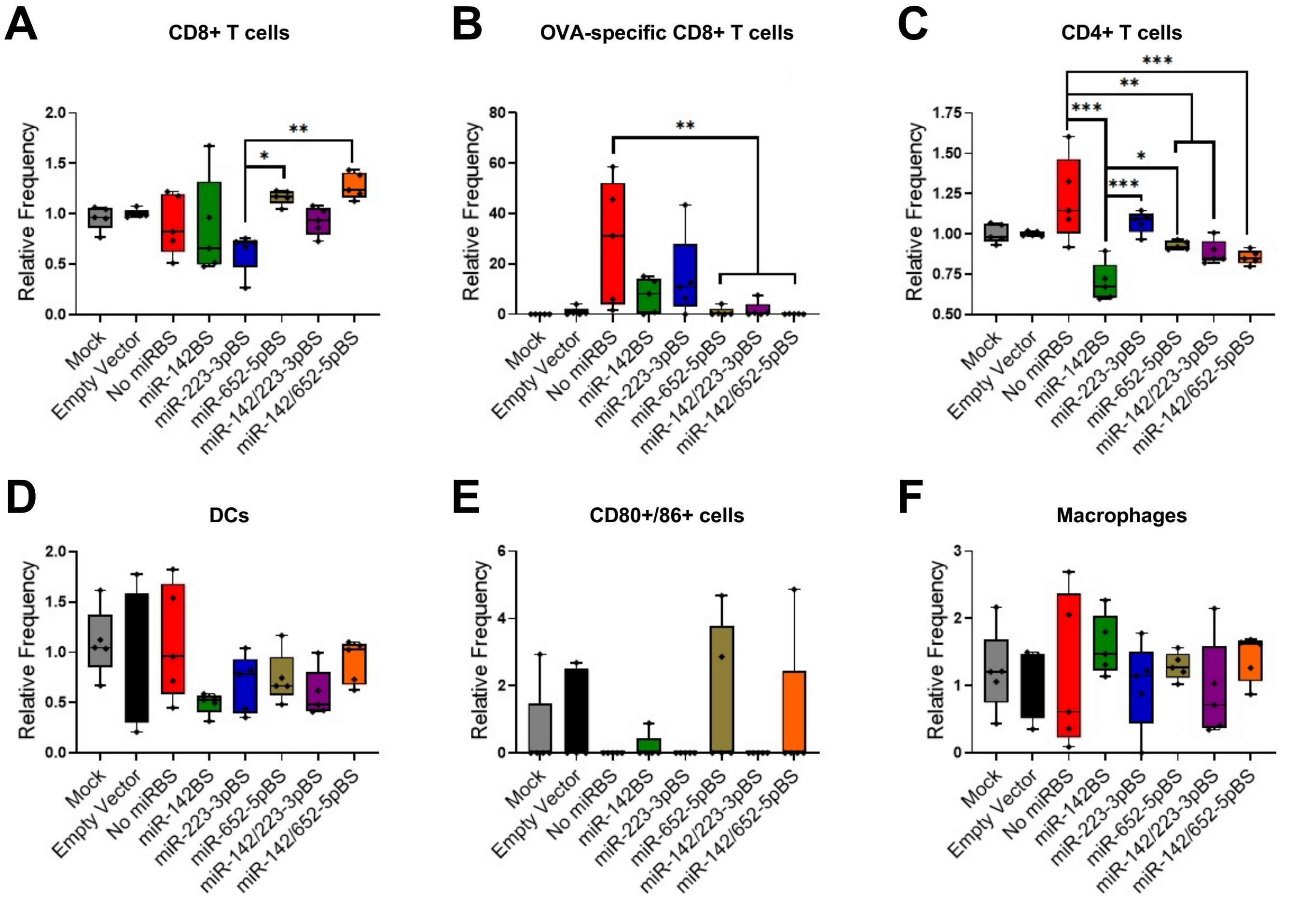

Supplementary Figure 5. miR-BSs do not affect activation status of immune cell populations in circulation. PBMCs were isolated from injected mice two weeks after treatment followed by staining for macrophages (**A**), DCs (**B**), activated DCs (**C**), CD8 T cells (**D**), OVA-specific CD8 T cells (**E**), and CD4 T cells (**F**). Flow cytometry data are displayed as relative frequencies were plotted as box plots with means, first and third quartile boundaries, and whiskers indicating max and min values (n = 5). p values were estimated by one-way ANOVA with Tukey's post hoc test. *$p < 0.05$, **$p < 0.01$, ***$p < 0.001$.

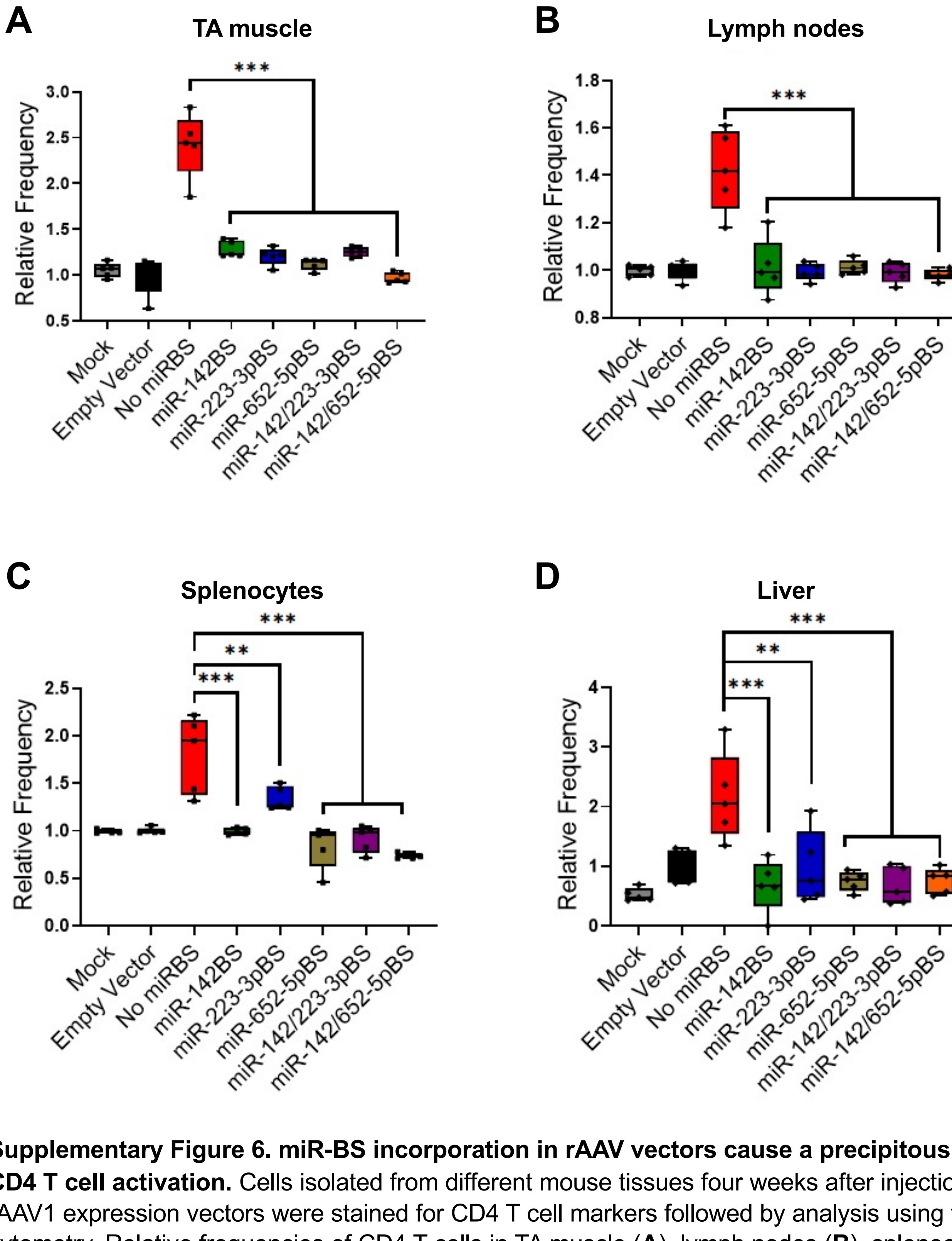

Supplementary Figure 6. miR-BS incorporation in rAAV vectors cause a precipitous drop in CD4 T cell activation. Cells isolated from different mouse tissues four weeks after injection of rAAV1 expression vectors were stained for CD4 T cell markers followed by analysis using flow cytometry. Relative frequencies of CD4 T cells in TA muscle (**A**), lymph nodes (**B**), splenocytes (**C**), and liver (**D**) are shown as box plots with means, first and third quartile boundaries, and whiskers indicating max and min values ($n = 5$). p values were estimated by one-way ANOVA with Tukey's post hoc test. *$p < 0.05$, **$p < 0.01$, ***$p < 0.001$.

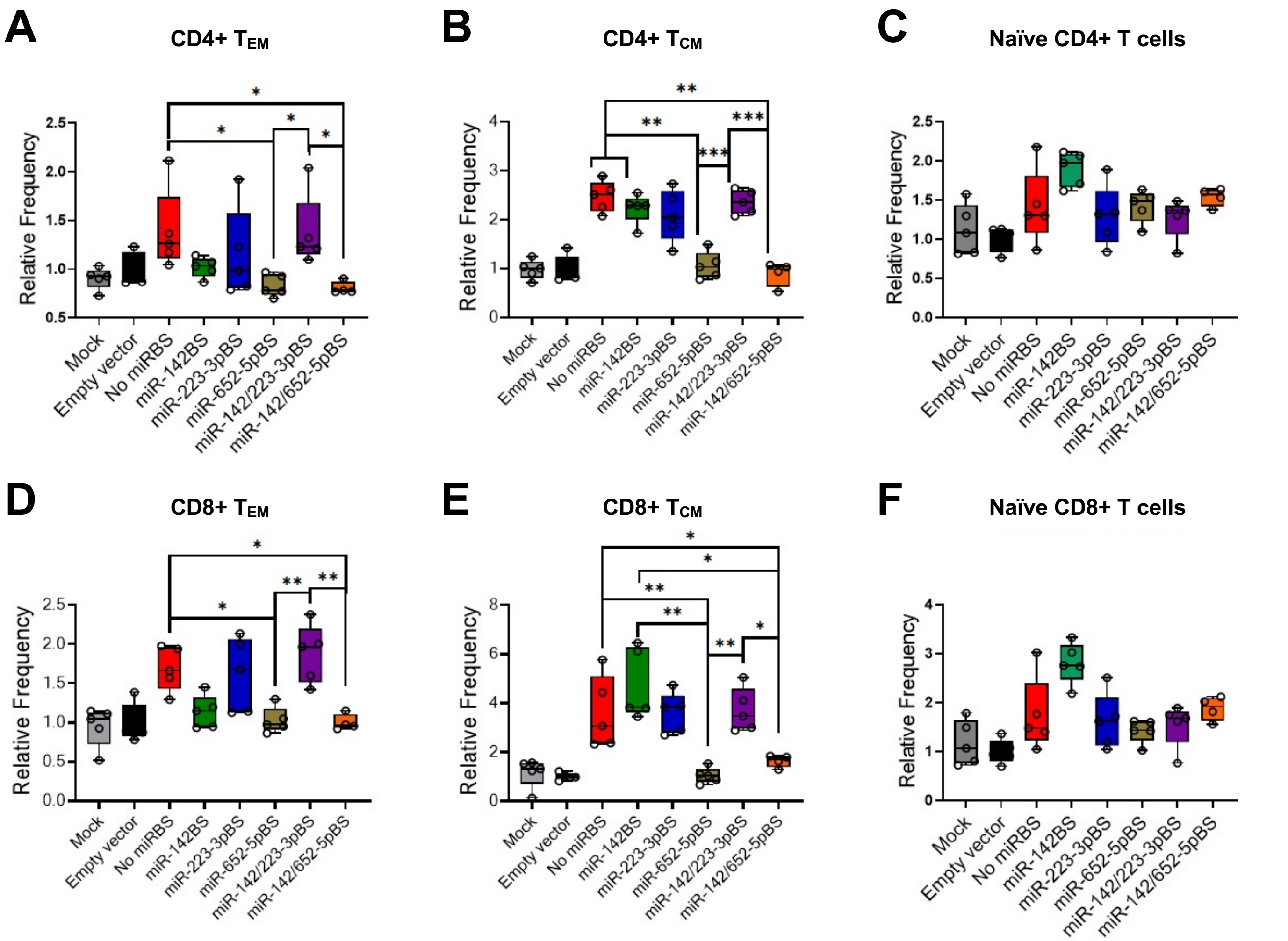

Supplementary Figure 7. miR-652-5pBS and miR-142/652-5pBS-containing vectors efficiently suppress OVA specific memory T cells. Splenocytes harvested from C57BL/6 mice 2-weeks post rAAV1 injection were stimulated with OVA (5 µg/mL) for 24 hours followed by staining with CD44 and CD62L to profile for both CD4+ and CD8+ effector memory (**A** and **D**; CD44^{high}CD62L^{neg}, T_{EM}), central memory (**B** and **E**; CD44^{high}CD62L+; T_{CM}) and naïve T cells (**C** and **F**; CD44^{low}CD62L+). Analysis was done by flow cytometry and relative frequencies are plotted as box plots with means, first and third quartile boundaries, and whiskers indicating max and min values (n = 5). p values were estimated by one-way ANOVA with Tukey's post hoc test. *$p < 0.05$, **$p < 0.01$, ***$p < 0.001$.

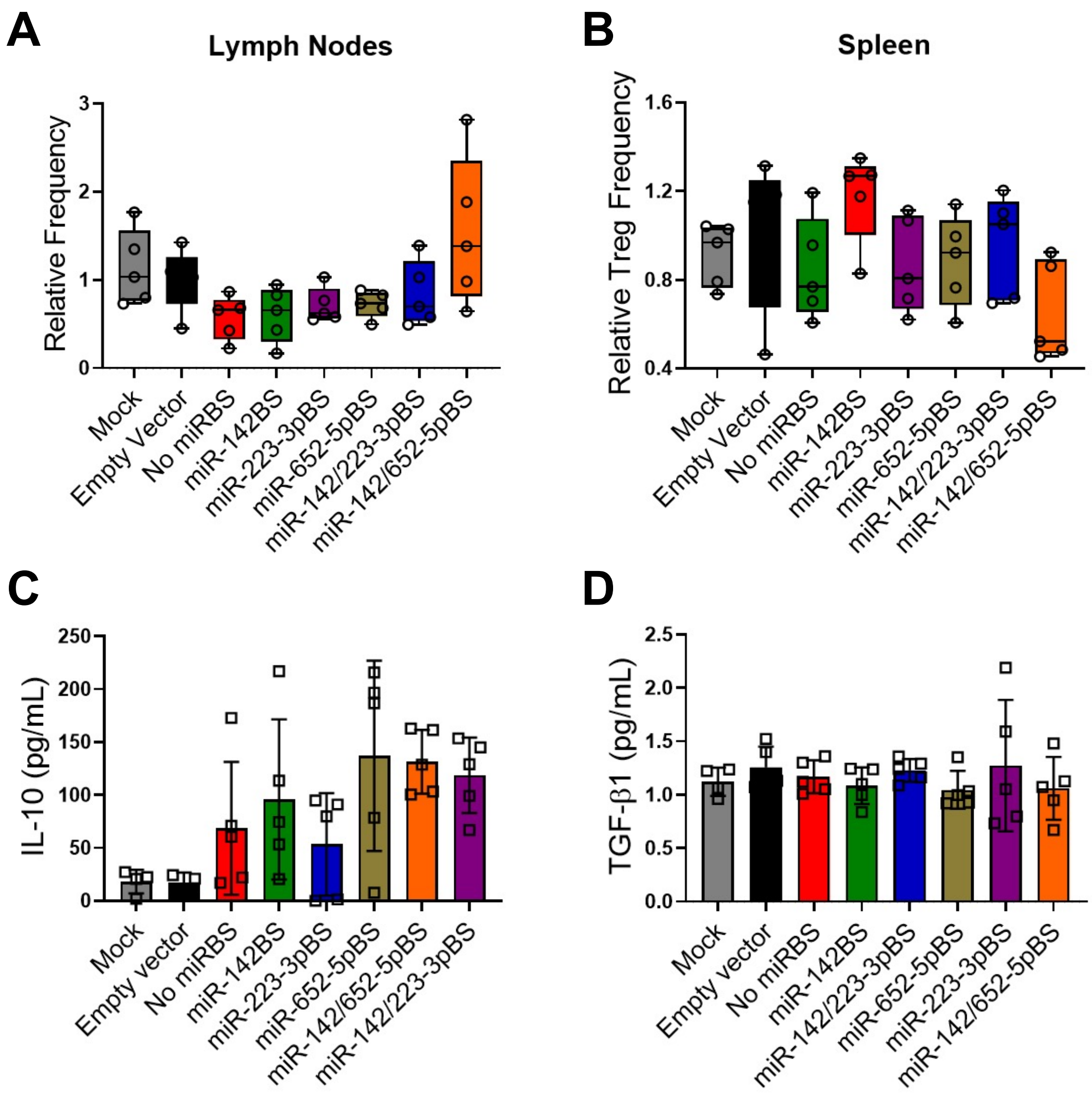

Supplementary Figure 8. Incorporation of miR-BSs has no effect on regulatory T cell populations. C57BL/6 mice were injected with rAAV1 vectors and cells from lymph nodes and spleens were isolated two weeks after treatment. Cells were stained for the expression of Treg markers and analyzed by flow cytometry. The relative frequencies are shown as box plots with means, first and third quartile boundaries, and whiskers indicating max and min values (n = 5) for lymph nodes (**A**) and spleen (**B**). The levels of IL-10 (**C**) and TGF-β (**D**) that were secreted by stimulated splenocytes four weeks after vector injection were estimated by ELISA (mean ± SD, n = 5).

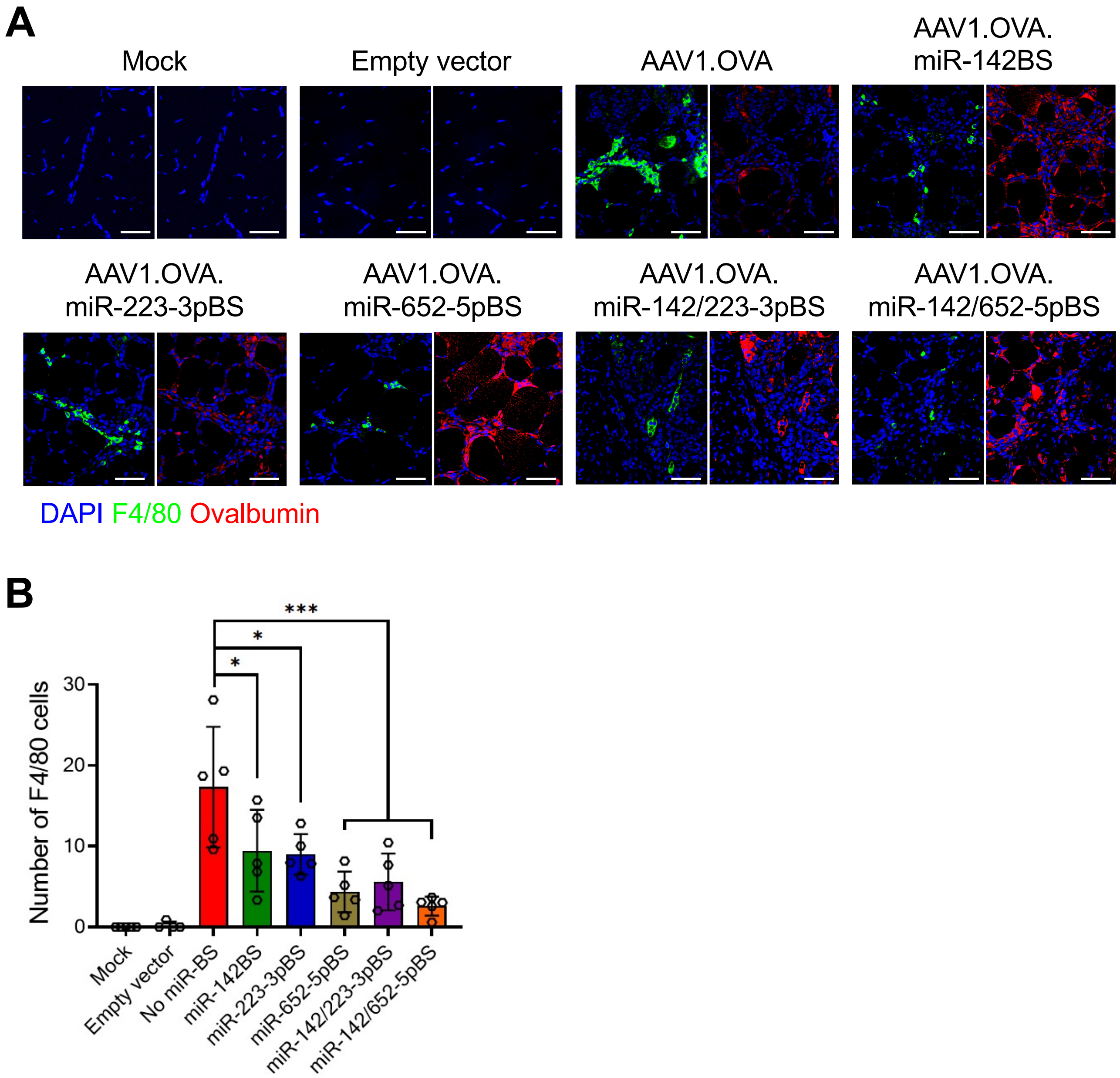

Supplementary Figure 9. miR-652-5p and miR-142/652-5pBS cassettes confer robust repression of macrophage activation and support stable OVA transgene expression. C57BL/6 male mice, six weeks old, were i.m. injected with rAAV1.*OVA* vectors with or without miR-BS (1×10^{11} GCs/mouse, $n = 5$) and sacrificed two weeks after injection to harvest injected TAs. (**A**) Immunohistochemistry staining to detect macrophages and transgene expression was performed by using antibodies against F4/80 (left panels) and OVA (right panels), respectively. Images were acquired at 40x magnification (DAPI, blue; anti-F4/80, green; and anti-OVA, red). Scale bars correspond to 50 μm. (**B**) Quantification of F4/80 images in four fields at original magnification of 40x ($n = 5$). p values were estimated by one-way ANOVA with Tukey's post hoc test. *$p < 0.05$, **$p < 0.01$, ***$p < 0.001$.

Supplementary Table 1: p-values from the one-way ANOVA with Tukey's multiple comparisons test of OVA levels between different injection groups

Tukey's multiple comparisons test	Adjusted p value
No miRBS vs. miR-142BS	<0.0001
No miRBS vs. miR-223-3pBS	<0.0001
No miRBS vs. miR-142/223-3pBS	<0.0001
No miRBS vs. miR-652-5pBS	<0.0001
No miRBS vs. miR-142/652-5pBS	<0.0001
No miRBS vs. Empty vector	0.9601
No miRBS vs. Mock	>0.9999
miR-142BS vs. miR-223-3pBS	0.9841
miR-142BS vs. miR-142/223-3pBS	0.9866
miR-142BS vs. miR-652-5pBS	0.3095
miR-142BS vs. miR-142/652-5pBS	0.0008
miR-142BS vs. Empty vector	<0.0001
miR-142BS vs. Mock	<0.0001
miR-223-3pBS vs. miR-142/223-3pBS	0.6469
miR-223-3pBS vs. miR-652-5pBS	0.0536
miR-223-3pBS vs. miR-142/652-5pBS	<0.0001
miR-223-3pBS vs. Empty vector	<0.0001
miR-223-3pBS vs. Mock	<0.0001
miR-142/223-3pBS vs. miR-652-5pBS	0.8185
miR-142/223-3pBS vs. miR-142/652-5pBS	0.0076
miR-142/223-3pBS vs. Empty vector	<0.0001
miR-142/223-3pBS vs. Mock	<0.0001
miR-652-5pBS vs. miR-142/652-5pBS	0.2076
miR-652-5pBS vs. Empty vector	<0.0001
miR-652-5pBS vs. Mock	<0.0001
miR-142/652-5pBS vs. Empty vector	<0.0001
miR-142/652-5pBS vs. Mock	<0.0001

Supplementary Table 2: p-values from the one-way ANOVA with Tukey's multiple comparisons test of anti-OVA levels between different injection groups

Tukey's multiple comparisons test	Adjusted p value
No miRBS vs. miR-142BS	<0.0001
No miRBS vs. miR-223-3pBS	<0.0001
No miRBS vs. miR-142/223-3pBS	<0.0001
No miRBS vs. miR-652-5pBS	<0.0001
No miRBS vs. miR-142/652-5pBS	<0.0001
No miRBS vs. Empty vector	<0.0001
No miRBS vs. Mock	<0.0001
miR-142BS vs. miR-223-3pBS	0.9991
miR-142BS vs. miR-142/223-3pBS	0.9926
miR-142BS vs. miR-652-5pBS	0.9999
miR-142BS vs. miR-142/652-5pBS	0.9577
miR-142BS vs. Empty vector	0.9585
miR-142BS vs. Mock	0.9585
miR-223-3pBS vs. miR-142/223-3pBS	>0.9999
miR-223-3pBS vs. miR-652-5pBS	>0.9999
miR-223-3pBS vs. miR-142/652-5pBS	0.7243
miR-223-3pBS vs. Empty vector	0.7267
miR-223-3pBS vs. Mock	0.7267
miR-142/223-3pBS vs. miR-652-5pBS	>0.9999
miR-142/223-3pBS vs. miR-142/652-5pBS	0.5852
miR-142/223-3pBS vs. Empty vector	0.5877
miR-142/223-3pBS vs. Mock	0.5877
miR-652-5pBS vs. miR-142/652-5pBS	0.8017
miR-652-5pBS vs. Empty vector	0.8037
miR-652-5pBS vs. Mock	0.8037
miR-142/652-5pBS vs. Empty vector	>0.9999
miR-142/652-5pBS vs. Mock	>0.9999

Supplementary Table 3: List of antibodies used for flow cytometry

Antigen	Company	Clone
F4/80	BioLegend	BM8
CD4	BioLegend	RM4-4
CD45	eBioscience	30-F11
CD80 (B7-1)	eBioscience	16-10A1
SIINFEKL	BioLegend	25-D1.16
CD3	eBioscience	17A2
CD86 (GL1)	eBioscience	GL1
CD8	eBioscience	53-6.7
CD11b	eBioscience	M1/70
CD11c	eBioscience	N418
CD44	BioLegend	IM7
IL17A	BioLegend	TC11-18H10.1
IFN-g	BioLegend	XMG1.2
CD62L	BioLegend	MEL-14
FOXP3	eBioscience	FJK-16s
